# Neandertal Demise: An Archaeological Analysis of the Modern Human Superiority Complex

**DOI:** 10.1371/journal.pone.0096424

**Published:** 2014-04-30

**Authors:** Paola Villa, Wil Roebroeks

**Affiliations:** 1 University of Colorado Museum, Boulder, Colorado, United States of America; 2 Unité Mixte de Recherche 5199, De la Préhistoire à l’Actuel, Culture, Environnement et Anthropologie (PACEA), Université Bordeaux 1, Talence, France; 3 School of Geography and Environmental Studies, University of the Witwatersrand, Johannesburg, South Africa; 4 Faculty of Archaeology, Leiden University, Leiden, The Netherlands; University of Oxford, United Kingdom

## Abstract

Neandertals are the best-studied of all extinct hominins, with a rich fossil record sampling hundreds of individuals, roughly dating from between 350,000 and 40,000 years ago. Their distinct fossil remains have been retrieved from Portugal in the west to the Altai area in central Asia in the east and from below the waters of the North Sea in the north to a series of caves in Israel in the south. Having thrived in Eurasia for more than 300,000 years, Neandertals vanished from the record around 40,000 years ago, when modern humans entered Europe. Modern humans are usually seen as superior in a wide range of domains, including weaponry and subsistence strategies, which would have led to the demise of Neandertals. This systematic review of the archaeological records of Neandertals and their modern human contemporaries finds no support for such interpretations, as the Neandertal archaeological record is not different enough to explain the demise in terms of inferiority in archaeologically visible domains. Instead, current genetic data suggest that complex processes of interbreeding and assimilation may have been responsible for the disappearance of the specific Neandertal morphology from the fossil record.

## Introduction

The demise of Neandertals is one of the most debated issues in paleoanthropology. Their disappearance in the fossil record constitutes the biological part of a process of change that occurred in Europe and in the Near East between approximately 45 and 35 thousand years ago (ka) [1]–[2]. In western Eurasia, the process led to the replacement of an archaic population (Neandertals) with Middle Paleolithic technologies by a population of modern humans (*Homo sapiens*) with Upper Paleolithic ones [3]–[5]. The study of this process of transition integrates data and scientists from a wide range of disciplines, including archaeologists, physical anthropologists, dating specialists, and increasingly so, geneticists.

Into the 1980’s many paleoanthropologists argued that the Neandertals had evolved into modern humans (or modern Europeans) and that the Upper Paleolithic derived from the Middle Paleolithic Neandertal culture. The opposite view assumed a single origin of modern humans and replacement of archaic populations, including Neandertals, by modern humans immigrating from an unknown source area [6]. This view became widely accepted with advances in genetic studies and dating of fossils and sites in Africa, Europe and the Near East. In 1987 the work of Cann and colleagues [7] provided compelling mitochondrial evidence for a recent African origin of all modern humans. Later, the genetic evidence was supported by fossils which showed that Africans were far more modern looking than their Neandertal contemporaries, with dates for the Omo Kibish 1 and Herto skulls in Ethiopia suggesting that the early modern human morphology emerged in East Africa possibly as early as 195,000 year ago [8]–[10]. There is now general agreement that modern humans originated in Africa, and subsequently expanded their range into the Near East and later into Europe. This is the core of the so-called Out-of-Africa hypothesis [11].

In tandem with these developments, archaeologists began looking for modern behavioral markers in African sites dated between 200,000 and 60,000 years ago. Many (see below) would now suggest that there is indeed evidence for significant behavioral and cognitive differences between Neandertals and their African contemporaries, and that when early moderns encountered Neandertals in Western Eurasia, these differences would have entailed the demise of the Neandertals.

### Hypotheses for the Demise of Neandertals

Virtually all explanations for the disappearance of the Neandertals from the Eurasian record point in one way or another to the arrival of *Homo sapiens*, anatomically modern humans (AMH), in Europe and western Asia. Late Pleistocene dispersal events brought AMH into the ranges of other hominin populations outside of Africa. In recent years we have seen a series of publications with detailed maps purported to show the progress of modern humans and their new technology across various Eurasian landscapes populated by “archaic” hominins, including Neandertals [12]–[15]. The source populations [16]–[19] and the routes supposedly taken by the advancing modern humans vary widely in these papers though, as do the chronologies for the supposed Out of Africa dispersal(s) of modern humans. Some archaeological estimates hypothesize an age of around 125 ka for the first AMH dispersals into the Arabian peninsula [20] and around 77 ka for India [21], while others suggest that AMH dispersal, thought to be associated with distinctively African technologies analogous to the “Howiesons Poort”, occurred only around 50–60 ka, i.e. after the Toba volcanic eruption at around 75 ka [22]. Genetic dates for the Out-of-Africa dispersal(s) of AMH also vary widely, between approximately 45 to 130 ka [23]–[25].

The disappearance of the archaic populations, including Neandertals, is routinely explained in terms of the “superiority” of modern humans, who had developed in Africa the ability to evolve complex cultural traditions and had become equipped with cognitive capacities which allowed them to expand globally and replace all other hominins [26], [27]. Such interpretations have increasingly become based on proxies in the Middle Stone Age (MSA) archaeological record of sub-Saharan Africa which, compared to the Middle Paleolithic record of Europe and western Asia, would testify to superiority in a wide range of domains, either in Africa and/or upon arrival of *Homo sapiens* in the Neandertal geographical ranges. These include inventiveness and capacity for innovation [11], [28], complex symbolic and linguistic abilities [29], [30], more efficient hunting strategies [31], exploitation of a broader range of resources including plants and aquatic ones [32], projectile technology [33]–[35], heat treatment of lithic raw materials [36], hafting technology [37], [38], planning capacities including larger scale social networks as shown by large transport distances of raw materials [39], environmental flexibility [40], memory capacity [41] as well as larger population sizes [42]. Inferiority in one or more of these domains has been at the core of many explanations for the demise of the Neandertals.

Prior to the last decade the cultural attributes listed above were generally considered as exclusive manifestations of the western Eurasian Upper Paleolithic, as the result of a major behavioral revolution compared to the preceding Middle Paleolithic. Seen from a European or Near Eastern perspective, the Upper Paleolithic witnessed the introduction of new technologies, the ability to communicate symbolically, systematic use of body ornaments and various forms of mobile and rock art, by modern humans expanding from Africa into Eurasia, leading to the gradual replacement of non-modern populations, such as the Neandertals [29], [43]–[45]. It was acknowledged that some of those features had emerged earlier in Africa, but the most complex technologies and art forms were seen as characteristic of the European Upper Paleolithic, thus clouding the issue of the source area where these innovations had taken place.

In 2000 McBrearty and Brooks [27] forcefully argued that the components of this “Upper Paleolithic revolution” were already visible in the African MSA, tens of thousands of year earlier. They suggested a gradual assembling of a package of modern human behavior in Africa, which was later exported to other regions of the Old World: a view contested by Klein [11], who stressed a later and punctuated emergence of “modern human behavior”. In 2003 D’Errico [46] reviewed the cultural attributes which McBrearty and Brooks saw as defining modernity. He argued that comparable traits also occur in the Neandertal record and rejected the theory that behavioral “modernity” indicators are uniquely associated with *Homo sapiens*. Nevertheless, the behavioral markers described by McBrearty and Brooks have in recent years increasingly been used to explain the demise of the Neandertals when modern humans expanded into their territories.

Non-archaeological data have also been called upon to explain the outcompeting of the large-bodied and big-brained Neandertals by modern humans, but these fall in first instance out of the scope of this review (but see Discussion). The goal of this paper is to test the strength of the archaeology-derived hypotheses for Neandertal extinction referred to above. While some of these hypotheses have been evaluated individually [47], ours is the first systematic study of a wide range explanations. It is timely too, given the large amount of new data generated by fieldwork in Africa, the resulting speculations on modern humans cognitive modernity [28], [30], and new insights into Neandertal behavior and biology, including their biological affinity with modern humans. Genetic studies now suggest that the debate on the demise of the Neandertals needs to be reframed in terms of some degree of interbreeding [23], [48], [49]. In that sense, Neandertals did not go extinct, even though their distinctive morphology did disappear. We will return to this topic at the end of this paper.

## Methods

Our evaluation of the key archaeology-derived explanations for the demise of the Neandertals entails a comparative study of the archaeological record of Neandertals and contemporary modern humans, i.e. AMH in Africa and Southwest Asia between 200 and 40 ka. To include younger periods would disregard the effects of cultural and technological evolution after the demise of the Neandertals. The various competing models regarding the evolutionary disadvantages of Neandertals are listed in Table 1 and are reviewed in detail in (Text S1 Hypotheses 1–11), where they are systematically described listing the specific hypothesis and supporting as well as refuting evidence.

**Table 1:** Hypotheses for the demise of Neandertals (a).

1. AMH had “complex symbolic communication systems” and “fully syntactic language”, while Neandertals did not.
2 Neandertals had limited capacity for innovations.
3. Neandertals were less efficient hunters.
4. Neandertal weaponry was inferior to AMH projectile technology.
5. Neandertals had a narrow diet, unsuccessful in competition with AMH with their more diverse diets.
6. The use of traps and snares to capture animals was the exclusive domain of AMH.
7. AMH had larger social networks.
8. The initial AMH populations entering Neandertal territory were significantly larger than regional Neandertal populations.
9. Hafting by AMH required complex procedures indicative of modern cognition, while Neandertals hafting was a simple procedure using naturally available glues.
10. Cold climate around 40 ka was a factor in Neandertal decline.
11. Eruption of Mount Toba volcano at 75 ka played an indirect role in Neandertal extinction.

(a) See Text S1 Hypotheses 1–11 for details.

### Transitional Industries

The so-called “transitional industries”, which show some similarities to late Middle Paleolithic (Mousterian) industries but also contain Upper Paleolithic forms and whose time range falls within a 45−35 ka interval, will not be discussed in detail here, for the following reasons:

- the makers of the Bohunician, Bachokirian, Szeletian and Streletskayan [in Central and Southeastern Europe and Russia) are not known yet (late Neandertals or AMH?) and hence their status is ambiguous [50].
- Neandertals are accepted by many–though not by all [51] - as the makers of the Châtelperronian, best known from the Grotte du Renne at Arcy-sur-Cure in France, excavated by Leroi-Gourhan and his team between 1949 and 1963 [52]. The interpretation of the industry, rich in distinctively “modern” cultural features such as ornaments and bone tools, has been the subject of heated debates. The controversies about whether the ornaments and bone tools were (i) an invention of Neandertals [46], [53], (ii) the result of stratigraphic admixture of Neandertal remains and Upper Paleolithic artifacts [54]–[57], or (iii) due to acculturation [28], [58], [59] have been going on since the acculturation hypothesis was most explicitly discussed in 1998 by D’Errico *et al.* [60]. The stratigraphic integrity of the Châtelperronian layers at the site has been reaffirmed in a recent paper [61], contra [62]. New radiocarbon dates of 44,970−44,520 cal BP for the start and 41,300−40,570 cal BP for the end of the Châtelperronian at Arcy and of 41,950−40,660 cal BP (all dates with probability at 68.2%) for the Saint Césaire Neandertal suggest that the makers of the Châtelperronian ornaments were indeed Neandertals [63]. However, the conflicting hypotheses of acculturation versus independent invention persist, as the dates appear to postdate or overlap in time with the arrival of early modern humans in Italy [5] and with dates for the Aurignacian in Germany [63; contra 2].

Early descriptions of Châtelperronian assemblages stressed a Mousterian component but the industry is now considered Upper Paleolithic in technology, although different from the Aurignacian, and the presence of Mousterian tools as due to syn-or post-depositional mixing [51]. The Châtelperronian lithic industry recently studied at the open-air site Canaule II in France [64] is also described as fully Upper Paleolithic, based on its technology and almost exclusive production of blades and backed points. In contrast to the Arcy site, the very large assemblage of Canaule II comes from a thin and unique layer, with its integrity and homogeneity confirmed by refitting. The absence of any Middle Paleolithic elements in this Châtelperronian assemblage again strongly suggests that the Châtelperronian, chronologically intermediate between the Middle and the Upper Paleolithic, is a unique entity, not the result of a mix of Middle and Upper Paleolithic artifacts.

1. The Uluzzian, an Italian transitional industry also present in Greece and previously attributed to Neandertals [60], [65], is now seen by some as a product of modern humans, on the basis of a study of two deciduous teeth from Grotta del Cavallo in southern Italy [5]. AMS dates on shell beads from Grotta del Cavallo yielded 45.010−43,380 cal BP for the lower Uluzzian layer. If the dates and the taxonomic attribution are accepted, they would extend the period of Neandertal-modern human coexistence to some millennia. Neandertals are thought to have persisted in southern Iberia until 37 ka, based on the dates for Middle Paleolithic assemblages there [66] and at other sites in Europe based on dates for Neandertal remains at Spy (Belgium) and Vindija (Croatia). Elsewhere the dates for the Campanian Ignimbrite ash horizon, stratigraphically above several Proto-Aurignacian layers, situate the end of the Middle Paleolithic at about 40 ka (see *The date of the demise*).

The “transitional” industries are extremely relevant to understand the routes of migrations and expansion of AMH in Europe, the nature of cultural contacts between the local and immigrant populations and the onset of the Upper Paleolithic in those regions. However, we need more contextual (i.e. stratigraphic, technological and in some cases fossil) data before we can make accurate assessments of the evidence, e.g. in terms of the type of hominin authorship [62]. In the case of the Châtelperronian, attributed to Neandertals by several scholars, we will review recent evidence from sites where stratigraphic admixture can be excluded and are less controversial than Grotte du Renne.

## Results

Explanations for the demise of Neandertals have been developed at various levels of abstraction, and include topics notoriously difficult to study in the archaeological record, such as “complex symbolic communication systems” [28], “fully syntactic language” [67] or “cognitive capacities” in general. Other hypotheses refer to behavioral domains which do leave clear traces in the archaeological record, provided the right taphonomic conditions prevail (Table 1; Text S1, Hypotheses 1–11).

### Language and Symbolism

The archaeological record has been mined in various ways to produce evidence for symbolic aspects of human culture, with a strong focus on the emergence of language. Archaeological finds from the MSA have been used to build scenarios for the timing and location of the origin(s) of language. Several of these finds come from South Africa and include engraved pieces of ochre from Blombos Cave [68]–[69], *Nassarius* shells from the same location [70], and heated silcrete artefacts from the site of Pinnacle Point, said to testify to sophisticated pyrotechnological know-how by early modern humans [36]. Botha has shown the assumptions and series of inferential steps some of these authors had to make before being able to squeeze “language” out of their mute artefacts [71]–[72], see also [73]–[74] pinpointing the weak spots in the steps leading from observations about archaeological phenomena to statements about the presence of “fully syntactical language”. Moreover, recent data on Neandertal use of ochre and manganese as well as on Neandertal production of pitch, the presence of transported and ochre-smeared shells, of ornaments such as eagle claws and perhaps bird feathers [75]–[78] (Text S1, Hypothesis 1), and the production of the specialized bone tools recently reported from two late Middle Paleolithic sites [79] (Text S3, Lissoirs) indicate no significant differences between the MSA data commonly used to create these more abstract explanations and the later Middle Paleolithic record.

The same applies to explanations regarding behavioral domains which do leave clear traces in the archaeological record. In our study none of the explanations listed in the introduction and in Table 1 proved to be supported by adequate archaeological data.

### Hunting Methods and Diet

With the demise of the idea that Neandertals were scavengers and ineffective hunters [80]–[82], the former interpretive framework has to some degree been reformulated in terms of Neandertals inferiority in subsistence strategies and hunting weaponry for which, again, there is no support from the archaeological record (Text SI, Hypotheses 3–4). Neandertals were by all means accomplished large game hunters, who survived in a wide range of environments subsisting by hunting a wide range of animals in a variety of topographical settings. In contrast to prevailing ideas [31], [83], their diet was not restricted to large and medium size herbivores only. Several sites document a broader diet, including aquatic foods, small fast game (birds, rabbits) as well as plant resources (*SI Hypothesis 5*). Likewise, the idea that spear throwers and bow and arrow were first developed in the MSA of South Africa before 60 ka and conferred substantive advantages on modern humans as they left Africa and encountered Neandertals equipped with only hand-cast spears [33], [84] may be correct, but there is no solid archaeological evidence in its support (Text S1, Hypothesis 4.3).

### Organized Use of Space

The same applies to purported differences in the use of space at the level of camp sites by AMH and Neandertals, with organized use of space seen as typical for AMH. The South African MSA record has some cases of excellent preservation of plant materials in dry conditions, including possible bedding material recovered from 77 to 58 ka old deposits at Sibudu [85]–[86]. Some researchers have taken the presence of bedding material and “the deliberate use and organization of living space” to be “an important trait of culturally modern behavior” [87]. However, there exists good evidence for well-delimited activity areas at Neandertal sites such as Kebara, Amud (Israel) and Tor Faraj (Jordan) as well as from several European sites where the task-specific areas are documented by refitting (Text S1, Hypothesis 3). Furthermore, bed building by great apes is a well-documented learned behavior, dependent on appropriate early experiences [88].

### Capacity for Innovation

Another prominent scenario suggests that the archaeological record of sub-Saharan modern humans, to wit of the two main technocomplexes of the South African late MSA, the Still Bay (SB) and the Howiesons Poort (HP), indicates very dynamic and innovative phases which lasted less than 10,000 years each [26], [89]. These would constitute a striking contrast to the record of the Neandertals, who supposedly lacked the capacities to innovate and “made the same kinds of tools for 200,000 years without ever tinkering with the basic components” [90]. Recently reported dates from the Diepkloof site (South Africa) are significantly complicating our views on cultural change in the Late Pleistocene there, however. According to these new dates [91]–[92] the SB and HP technocomplexes would have a much longer duration than previously envisaged [89], comparable to those of broadly contemporaneous Middle Paleolithic industries in Europe, which show clear spatio-temporal distributions (Table 2, Text S1 Hypothesis 2). Jacobs’ OSL age estimates for the SB and HP are considered controversial by some [93]. More dating work is clearly required, while systematic technological and typological analyses are necessary to dispel doubts about assemblage definition, especially for the MIS 5 occurrences.

**Table 2:** Dates of technological phases in the late Middle Paleolithic of Europe and in the late Middle Stone Age of South Africa (a).

Technological phases	Start (ka)	End (ka)
**Still Bay**		
Blombos	ca.75.5 (OSL)	67.8 (OSL)
Sibudu	70.5±2.0 (OSL)	
Diepkloof	109±10 (TL)	
**Howiesons Poort**		
Sibudu	64.7±2.3 (OSL)	61.7±2 (OSL)
Klasies River Main Site, Cave 1A	64.1±2.6 (OSL)	56±3 (TL)
Border Cave	74±4 (ESR)	60±3 (ESR)
Diepkloof	105±10 (TL)	52.5±5 (TL)
**Post-Howiesons Poort/MSA III** (b)		
Klasies, Cave 1A	60±5 (TL)	–
Klasies, Cave 1A	57.9±5.3 (OSL)	–
Border Cave	60±3 (ESR)	44−42 (^14^ C cal BP)
Sibudu	58.5±1.4 (OSL)	38.6±1.9 (OSL)
Boomplaas	ca 56±6 (U-series)	38−36 (^14^ C cal BP)
Klein Kliphuis	57.8±2.4 (OSL)	33.3±1.3 (OSL)
Rose Cottage	56.0±2.3 (OSL)	–
**Mousterian in Western Europe** (c)		
Mousterian of Acheulian Tradition(six sites in SW France)	70	40
Quina Mousterian(six sites in SW France)	73	40
The *Keilmessergruppen*(13 sites in Germany, Poland and the Czech Republic)	80	50

(a) After [89], [91]–[92], [94], [96]–[105]. We have excluded assemblages with uncertain stratigraphy (Umhlatuzana, HP layers at Klein Kliphuis) or unpublished dates (Hollow Rock Shelter).

(b) The term Post-Howiesons Poort is equivalent to MSA III at Klasies River Main Site. It includes informal designations of the Sibudu sequence such as late MSA and final MSA. We have not included several TL and OSL dates for the HP and Post-HP of Rose Cottage because they are inconsistent or only informative for the middle part of the sequence [105]–[106]. The Post-HP OSL date reported here for Rose Cottage [89] is of layer LIN which is toward the base of the Post-HP sequence but above its oldest layer.

(c) The Middle Paleolithic technocomplexes are dated by TL, ESR, ^14^C (calibrated BP) and chronostratigraphy.

In contrast it is clear that the Post-HP technocomplex, characterized by unifacial points on flakes (Sibudu) or blades (Border Cave, Klasies), hard hammer percussion, rare presence of the Levallois technique and of formal tools on blades (Klasies) and flakes (esp. Rose Cottage and Sibudu), has a duration of about 20,000 years; even more if “transitional” or late MSA sites in South Africa, dated between 40 and 20 ka, are taken into account. These include three layers at Rose Cottage, dated between ca 30.8 and 27 ka, and Strathalan Cave B, with two layers dated between 29 and 25.7 ka [94]. OSL and ESR dates for post-HP assemblages are supported by AMS radiocarbon dates. Thus the pace of change and the evolutionary patterns of the European Upper Pleistocene record, which shows regional differentiation, cultural traditions and technological changes through time, are comparable to what is known from the African record. Technological and tool-type changes in the Mousterian industries precede by far the advent of Proto-Aurignacian and Aurignacian industries. Whatever dates are accepted for these industries [95], changes in Mousterian industries occurred long before 50 ka.

### Size of Social Networks

Other workers have suggested that Neandertals and AMH differed significantly in the sizes of their social networks. AMH larger-scale social networks are supposed to have acted as a buffer against environmental downturns, thus fostering long term survival. Such inferences are based on the translation of distances over which artifacts were transported in the deep past into statements about former mobility strategies, exchange systems and sizes of social networks. Yet it is almost impossible to differentiate between long distance transport as a signature of direct procurement as opposed to indirect acquisition, such as through trade or exchange networks [107]. Our review of the evidence (Text S1, Hypothesis 7) shows that as far as the archaeological record for raw material transfer distances is concerned, the MSA and the Middle Paleolithic record are not significantly different, despite of the obvious ecological differences between western Eurasia and Africa.

### Hafting Procedures, Heat Treatment and Cognition

According to another hypothesis Neandertals hafting of tools was a simple procedure, only using naturally available glues. Early modern human hafting techniques entailed complex procedures which required “abstract reasoning” and are hence indicative of modern cognition. According to Wynn and Coolidge [41] evidence for complex hafting procedures dates back to about 70 ka in South Africa. Replication experiments suggest that HP hunters used a mixture of plant gum, beeswax and powdered ochre to produce an adhesive that had to be carefully dried using fire [37]. However, from 200,000 years ago onward, European Neandertals used fire to synthesize pitch from bark, through a process that involved distillation in the absence of oxygen and within a temperature interval of 340°C–400°C [108]. Pitch is not a naturally occurring glue; it is a man-made material produced using fire as a tool. Birch park pitches have been experimentally produced in small dug out and subsequently covered pits beneath camp fires [109], though in very small quantities only, leaving open the question how exactly Neandertals produced their pitches. Two flakes associated with elephant remains at the Italian site of Campitello (Tuscany, Italy) were found enclosed in blackish organic material that was analyzed by gas chromatography/mass spectrometry and identified as a pitch obtained by a pyrolysis-type process of birch bark for hafting the flint flakes [110]–[111]. The Campitello finds date to the end of MIS 7. Comparable finds of birch bark pitch come from the German site Königsaue A, with an estimated age of 80 ka [112]–[113]. On basis of the stratigraphy of the site, the AMS dates of 43,800±2100 BP and 48,400±3700 BP cited in ref [113] should be considered minimum ages. Mania’s fieldwork at the site produced two pieces of pitch, one with fingerprints as well as the imprint of a stone tool and a wooden haft. Experimental studies show that production of pitch in the absence of air-tight pottery containers requires a high degree of technical knowledge.

According to Brown et al [36] heat treatment of silcrete at the South African site of Pinnacle Point at c. 72 ka and possibly as early as 164 ka indicates sophisticated knowledge of fire and elevated cognitive abilities that may have been a behavioral advantage on Neandertals as early modern humans moved to Eurasia. The evidence of pitch production as early as 200 ka by European Neandertals shows that those “elevated cognitive abilities” were not the exclusive domain of modern humans.

The straightforward scenario of superior AMH moving into Neandertal territory is also complicated by the Late Pleistocene occupation history of the East Mediterranean Levant. AMH were present in that region between 80 and 130 ka, and created the Skuhl and Qafzeh record with its burials, pigments and personal ornaments [114], associated with a Middle Paleolithic lithic technology. Between 80 and 47 ka however, only Neandertals are known from the fossil record of the Levant [115]. If the absence of fossil AMH in the record represents a true absence from the region, this could indicate that the Skuhl/Qafzeh hominins and their immediate descendants indeed may have “lacked the behavioral capacities that enabled subsequent modern humans to compete successfully against the Neanderthals” [115].

## Discussion

We conclude that all the “archaeology-based” explanations for the demise of the Neandertals reviewed here (Table 1, Text S1, Hypotheses 1–11) are flawed. They were based on much less data than we have available today and were at least in part the result of a long tradition of thinking in terms of Neandertals-AMH dichotomies, steered by overstressing developments within the Upper Paleolithic of Europe, the record of which has become almost like a yardstick for modern human behavior (Text S2).

While the debate about AMH dispersal times and routes out of Africa is intense, based on a range of archaeological as well as genetic data, the archaeological record from the various continents does not provide strong support for any of the suggested routes nor any of the suggested factors in the demise of the Neandertals. The very fact that the migration time estimates vary so widely suggests that we simply have no solid data; perhaps there was more than one migration event (in addition to the Last Interglacial limited expansion in the Levant), and in all probability the migrating groups did not have a strong cultural homogeneity. This may explain why we do not see clear archaeological signatures for AMH on the move.

Interestingly, the widely accepted date of 60 or 50 ka for the modern human expansion into Eurasia (following the earlier short-lived exodus in the Levant documented at Skuhl and Qafzeh) would rule out South Africa as the location for source populations for two reasons: (i) by 60 ka the HP tradition of backed tools made on blades and bladelets produced by soft stone hammer (supposedly associated with the AMH expansion) had given way to the post-HP assemblages characterized by a variety of flake tools and blades produced by hard hammer percussion but without backed blades [94], [103], [105], [116]; (ii) the Still Bay and HP populations were not larger than other MSA populations and might even have been smaller, thus excluding population pressure as the prime mover of the migration [117]. According to Klein [11] the Out of Africa expansion was underlain by a neural mutation that promoted the final development of the modern human brain. Direct evidence for this hypothesis may come from comparisons of Neandertal and modern human genomes.

In the recent past, much debate has been generated from the observation that Neandertals began to produce a richer archaeological record, including bone tools, personal ornaments and use of manganese and ochre, at the time when AMH started colonizing Europe. Some interpreted this change in the record as the result of Neandertal absorption of ideas and techniques from the incoming AMH. After having produced a rather monotonous record for almost 300,000 years, an independent invention of these new items just at the time of the arrival of AMH would have to be seen as an “impossible coincidence” [28]. However, as reviewed here, use of ochre, of personal ornaments, production of specialized bone tools and complex hafting techniques were part of the Neandertal repertoire already before the arrival of AMH in western Eurasia.

The present review also suggests that some of the innovative technologies of the Protoaurignacian and of the Aurignacian may have developed out of a Middle Paleolithic base (for a similar viewpoint, see [118]). Some components that occur sporadically or episodically in Neandertal and late MSA assemblages become much more common later, like pigment use, symbolic objects, extensive transport of raw materials and even specialized bone tools [79]. The same goes for another element, the intentional production of bladelets (<4 cm in length) from bladelet cores. Bladelets have been considered a discriminant factor between the Upper and Middle Paleolithic and therefore between AMH and Neandertals [119]. Production of bladelets has been securely identified in French Mousterian assemblages, e.g. at Combe Grenal (layers 30–29 and layers 16 and 14), Champ Grand and Grotte Mandrin, and in Spain at sites such as El Castillo and Cueva Morin [120], [121]. All these assemblages belong to the final Mousterian, with the exception of Combe Grenal and Grotte Mandrin; at the latter site, a layer with blades, bladelets and microlithic points is overlain by five layers with flake-based Mousterian assemblages [121]. At Combe Grenal layers 29–30 have an estimated age of late MIS 4, i.e. around 60 ka. Bladelets and bladelet cores are not abundant (5% of the assemblage at Combe Grenal layers 29–30), yet they show that Neandertals, like late MSA humans and the makers of the Protoaurignacian, mastered the technology of bladelet production, albeit using methods different from the HP small blade technology. It is their frequency, not cognition or technical competence, that distinguishes AMH bladelet production from that of Neandertals [120]. The techniques and methods of bladelet making in the Mousterian are different from those of the Protoaurignacian, just as the kind of possible symbolic objects are also different (use of raptor claws; on perforated or grooved animal teeth (see Text SI, Hypothesis 1). Perhaps the nature of the contacts should be seen in terms of diffusion of ideas rather than as face to face interaction and the copying of specific objects [122]. The occurrence of Dufour bladelets (often used as projectile elements in the Aurignacian and the Protoaurignacian) with very specific techniques of manufacture in the Châtelperronian of Quincay is interpreted in a similar way, as a form of low-degree social interaction between Neandertals and modern humans [123].

### The Date of the Demise

Various new dates support the idea of some chronological overlap between AMH and Neandertals, which may have enabled interbreeding and cultural interaction in western Europe: AMS dates on ultrafiltered bone collagen from the Châtelperronian layers X and IX of Grotte du Renne at Arcy, c. 44 to 41 kyr cal BP; the date of the Saint-Césaire Neandertal at 41.9−40.6 kyr cal BP [63]; the fact that the Protoaurignacian at the Italian sites of Castelcivita and Serino is overlain by the Campanian Ignimbrite tephra, dated to 39.28±0.11 ka by ^40^Ar/^39^Ar [124]; the modeled age ranges of c. 41.5−39.9 kyr cal BP of several radiocarbon-dated Proto-Aurignacian sites [95]; the date of the Oase 2 early modern cranium at c. 40 ka [125]; the AMS dates for the Neandertal child from Spy cave (Belgium), 36,870 to 38,494 and 37,297 to 40,490 cal BP [126]; the AMS dates for the Vindija (Croatia) Neandertal remains at c. 38 kyr cal BP [127], [128]; the 37.4 ka cal BP date for the final Mousterian level of Cueva Antón in southeastern Spain [75]. Even if we do not consider dates judged by some as controversial such as (i) the AMS dates on shell beads for the layer containing the modern human teeth at Grotta del Cavallo at 45.010−43,380 cal BP [5, contra 2]; (ii) the dates for the Kent’s Cavern modern human maxilla [3, contra 2, 129]; and (iii) the dates for the Aurignacian at Geissenklösterle at c. 42 kyr cal BP [4, contra 2], some millennia of overlap are indicated The latest Neandertal currently known from the Levant is the adult male skeleton from Amud Cave (Israel) with an ESR date of 53±8 ka on tooth enamel [130].

### Interbreeding and Assimilation

For some authors replacement and supposedly rapid extinction of Neandertals can be explained only in terms of substantial cognitive, technological and demographic differences between the Neandertals and AMH [42], [131]. But, as we tried to show here, the Neandertal archaeological record was not different enough to explain their demise in terms of inferiority in archaeologically visible domains. Thus, if Neandertals were not technologically and cognitively “disadvantaged”, how can we explain that they did not survive?

Some modern human-like anatomical characteristics are said to occur in late Neandertal fossils (as in the Vindja, St. Césaire and Riparo Mezzena late Neandertals [132], [133] and refs therein) and vice versa some Neandertal features are present in early specimens of modern humans in Europe [134], [135] supporting a hypothesis of some degree of admixture between the two groups. However, until recently the morphological evidence of admixture was often dismissed. In 2010 a draft sequence of the Neandertal nuclear DNA provided clear evidence of interbreeding between Neandertals and modern humans [48], estimating that Neandertal inheritance makes up 1–4% of the genomes of people outside of Africa. A revised estimate based on a high-coverage sequence of a Neandertal from the Altai Mountains now suggests 1.5–2.1% [49]. Genes of Neandertals may have been favored through natural selection, and possibly played a role in the development of the immune system of modern humans [136] or in UV-light adaptations [137]. According to [138] gene flow from Neandertals to modern humans occurred between 47,000 and 65,000 years ago, and most likely happened at the time when Neandertals and modern humans encountered each other in Europe and the Middle East around 50,000 years ago.

In sum, interbreeding and assimilation, the tenants of a model first proposed by Fred Smith [139] are now supported by genetic data [134], [140]. It can be argued that the level of interbreeding may have been too limited to support an assimilation scenario. An interestingly parallel to this complex situation can be found in another “revolution”, the so-called Neolithic Revolution [43], [141], which does not feature explanations in terms of “cognitive” differences. The first farmers swept into Europe from the Near East at about 7500y BP displacing the local Late Mesolithic hunter-gatherers. But the Mesolithic hunter-gatherers, who cannot be described as cognitively inferior, were not submerged by hordes of farmers. Farmers and foragers coexisted for thousands of years in NW Europe; in Central Europe local hunter-gatherers adopted farming but in southern Scandinavia local foragers retained the Mesolithic lifestyle for c. 1500 years after farming arrived in Central Europe [142], [143]. Cultural contact is suggested by clear continuities in flint technology between the Mesolithic and early Neolithic in the region. After a very complex pattern of expansions and genetic shifts of the last 8,000 years the hunter-gatherer mitochondrial DNA haplogroups form 16% of the present-day Central European genetic composition [143]. It would take at least one millennium between the first arrival of immigrants and a notable increase in their population size.

The original Neandertal contribution to modern human biology may have been larger 40,000 years ago - equivalent to 2000 generations (with generation time at 20 years) – than estimates based on genomic regions of present-day humans suggest [144], [145]. Interbreeding of Neandertals and modern humans may have helped modern humans to adapt to non-African environments but also introduced alleles that were not tolerated and contributed to male hybrid sterility thus reducing the proportion of Neandertal ancestry of the period of contact to that seen today [144].

Mitochondrial genetic diversity of eight early modern European humans dated to ca 38,000 to 4,500 (^14^C cal BP, from Kostienki 14 to Őtzi The Iceman) is 1.5 times higher than that of five European Neandertals spanning the time to 38 to 70 ka [23], [146]. The high coverage genome of the Altai Neandertal [49] also suggests low genetic diversity which could indicate small population sizes (see Text S1 Hypothesis 8 for archaeological data). These genetic data suggest that differences in population sizes between the “resident” Neandertals and incoming AMH populations may have been a contributing factor in the absorption of Neandertal populations [23]. The momentous cultural changes that followed the arrival of AMH in Western Eurasia were not uniquely due to the residents’ cognitive or technological inferiority causing rapid and total replacement. The Neandertal demise appears to have resulted from a complex and protracted process [147] including multiple dynamic factors such as low population density, interbreeding with some cultural contact, possible male hybrid sterility and contraction in geographic distribution [148] followed by genetic swamping and assimilation by the increasing numbers of modern immigrants.

## Conclusion

In a review of the MSA and Middle Paleolithic archaeological record we have shown that inferred markers of modern human cognitive and behavioral capacities have a greater time depth in the Middle Palaeolithic record than commonly acknowledged. We have found no data in support of the supposed technological, social and cognitive inferiority of Neandertals compared to their AMH contemporaries. The results of our study imply that single-factor explanations for the disappearance of the Neandertals are not warranted any more, and that their demise was clearly more complex than many archaeology-based scenarios of “cognitive inferiority” reviewed here seem to suggest. This has implications beyond the field of archaeology per se: archaeologists’ characterizations of Neandertals as cognitively inferior to modern humans [149] have created an interpretive framework within which subtle biological differences between Neandertals and modern humans tend to be overinterpreted (see for instance [150].

After 40,000 years and 2000 generations the Neandertal fraction in non-African modern human genomes still constitutes a substantial legacy from these ancient hominins who differed from contemporary AMHs in both geno- and phenotypes [151] but whose archeological record was not different enough to support the purported cognitive “gap” between them and their contemporary modern humans.

## Supporting Information

Text S1 Hypotheses 1–11. Data on the various competing models on the evolutionary disadvantages of Neandertals, presented as Hypotheses 1 to 11, systematically described listing each specific hypothesis and supporting as well as refuting evidence.
(DOC)

Text S2 A single package of modern behavior?
(DOC)

Text S3 Lissoirs.
(DOC)
