# Supplementary material for: Neandertal Demise: An Archaeological Analysis of the Modern Human Superiority Complex
Source: PLoS One. 2014 Apr 30;9(4):e96424. doi: 10.1371/journal.pone.0096424 (PMC4005592; doi:10.1371/journal.pone.0096424)
Supplement: Text S1 — Hypotheses 1–11. Data on the various competing models on the evolutionary disadvantages of Neandertals, presented as Hypotheses 1 to 11, systematically described listing each specific hypothesis and supporting as well as refuting evidence. (DOC) [file pone.0096424.s001.doc]

**Neandertal Demise: An Archaeological Analysis of the Modern Human Superiority Complex**

Paola Villa, Wil Roebroeks

**Supporting Information**

**Text S1. Hypotheses 1-11.**

1. **AMH had complex symbolic communication system and fully syntactic language while Neandertals did not**.

***Hypothesis***. In 1989 Paul Mellars ] wrote a comprehensive review of facts and ideas, current at the time, on links between modern behavior and the emergence of complex forms of symbolism and language in the Upper Paleolithic. He noted the occurrence of coloring materials such as ochre and manganese, some reportedly showing scraping or facets due to use, at Mousterian sites in southwestern France [eleven Mousterian sites with pieces of manganese or ochre were listed by Demars ] for a total of 37 layers) and the occasional specimens of transported fossil shells [3, see also 4]. However neither pigments nor fossils were documented in any detail. Mellars also noted a few doubtful examples of non-utilitarian objects such as perforated bones (i.e. “pendants”) and bones and stones with supposed engravings. He emphasized the contrast between these rather ambiguous cases of symbolic artifacts and the large numbers of clearly man-made ornaments and figurative art documented since the early stages of the Aurignacian.

Aware of the limitations of the data, but aiming at a general explanation, he concluded that evidence of clearly symbolic behavior amongst Neandertals was sparse, and that they may have possessed some form of language although less complex than that documented for anatomically modern humans. The emergence of complex language and other forms of symbolic communications, in addition to more advanced technology, gave modern humans a crucial adaptive advantage and led to their successful dispersal across Eurasia. This remains a commonly accepted explanatory model for the Neandertal demise, although the cause and tempo of this behavioral shift remains controversial .

***Supporting evidence***. Many of the controversial symbolic artifacts mentioned by Mellars have now been shown to be natural. This is the case of the “engraved” rib from the MIS 6 layer of Pech de l’Azé II (France) of eleven bones with meandering marks from Cueva Morín (Spain), of grooves on an elephant vertebra from Stránská Skála (Czech Republic), and of perforated bone and phalanges, supposedly “pendants”, from Pech de l’Azé II, Kulna (Czech Republic) Bois Roche (France) and Bocksteinschmiede (Germany) The Divje Babe flute has been shown to be carnivore damage .

In contrast, archeological finds from the South African record have been used as building blocks for scenarios on the timing and location(s) of the origin(s) of language, suggesting that the anatomically modern southern African populations had fully modern cognitive abilities and syntactical language 75,000 years ago . These finds include engraved pieces of ochre from Blombos Cave, South Africa , *Nassarius* shells from the same location , and heated silcrete artefacts thought to testify to sophisticated pyrotechnological know-how by early modern humans in South Africa .

However, connecting archaeological objects to inferences regarding cognitive abilities and the possible presence of “fully syntactical” language remains a very controversial issue. In a dissection of the conceptual anatomy of such bridging attempts, Botha has shown the (often implicit) assumptions and series of inferential steps archaeologists have to make before being able to squeeze “language” out of mute artefacts , stressing the weak spots in those arguments. However, the engravings on the ostrich eggshell containers documented in the Howiesons Poort of Diepkloof indicate the existence of a graphic tradition of communicating among members of the MSA groups inhabiting the site.

***Refuting evidence***. A recent analysis of the microstructure and bio-mechanical performance of the hyoid bone from the 60,000 years old Neandertal skeleton at Kebara , concludes that this hyoid is not only similar to those of modern humans, as already suggested in 1989 , but also suggests that it was used in a very similar way. The hyoid bone plays an active role in speech, by providing support for the larynx and the tongue, and it seems that Neandertals were capable of speech. But did they possess the “the critical thought and syntactic ability necessary for complex language?” . Modern human language is syntactical, that is it consists of discrete components that can be combined to produce an almost unlimited number of sentences to communicate about objects, events and ideas ]. Given the difficulties of linking speech to language, we prefer to refrain from speculation about the evolution of language *per se* and provide instead well-documented recent evidence of Neandertal non-utilitarian, symbolic behavior, comparable to the contemporary MSA record. For a similar approach see d’Errico et al. and Dediu and Levinson ]. We review here recent research on possible pigments and objects which may have been used for personal decoration. The minerals from the 11 Mousterian sites cited by Demars ] for a total of 37 layers remain to be verified by modern analyses; of these sites only two, Pech de l’Aze 1 and IV (France), have been documented by recent research (see below).

*Pigments and objects for personal decoration.*

250 pieces of manganese with wear-facets and scraping documented by microscopic analysis have been found in layer 4 (Mousterian of Acheulian Tradition) at the site of Pech de l’Azé I. Based on OSL dating of sediments, layer 4 has a weighted mean age of 51.4 ± 2.0 ka . The site of Pech de l’Azé IV has yielded 26 pieces of manganese, of which 15 bear traces of modification . Several pieces of manganese with traces of being worked come from the Abri Peyrony in France ]. The interpretation of manganese as a coloring material is not as sure as that of ochre due to the scarcity of ethnographic and other archaeological sources on the use of manganese as a pigment. It does, however, find some support in the documented use of manganese oxides in Upper Paleolithic cave paintings ]. Given its flammable qualities, the use of manganese dioxide powder to rekindle or maintain a fire cannot be excluded [31].

At the MIS 3 open-air site of Les Bossats in Northern France there are 83 pieces of red ochre with multiple striations and scraping marks resulting from extracting red powder . Further evidence for Neandertal use of pigments comes from the finds of three perforated marine shells from Cueva de los Aviones (southeast Spain); one of the shells had stains of a red colorant identified as hematite around the perforation. A *Spondylus* shell contained residues of red and black pigments mixed with charcoal and pyrite, suggesting use as a paint container. Lumps of red and yellow colorants were found during the excavation; the sources of the material are located 3 to 7 km from the site. The Neandertal occupation is dated at between 45 and 50 ka. At the time of occupation the site was at some distance from the shore, about 1.5 to 7 km (the coast is in a subsidence area). There is also abundant evidence of the use of mollusks as food; the number of identified specimens is 785.

At Cueva Antón, in the same region but 60 km inland, level I-k yielded a perforated upper valve of *Pecten maxima* (scallop shell) which on its external side was covered with a mixture of yellow goethite and red hematite (sources for which are about 5 km from the site). The perforation may be natural but the hole existed before the application of colorant. The level was radiocarbon dated to 37-38 ka cal BP , but more recent excavations confirm that the small lithic assemblage represents a Middle Paleolithic occupation. While there is no clear evidence that the holes in the shells were anthropogenic, the authors conclude that Neandertals selected beached shells with holes 4.5-6.5 mm in diameter, convenient for stringing.

At the cave of Fumane (northern Italy) a Mousterian layer dated between 47.6 to 44.8 ka cal BP has yielded a marine shell (*Aspa marginata*) collected by Neandertals at a fossil exposure more than 100 km from the site. The shell outer surface was smeared with ochre, suggesting use as a pendant . Fragments of non-local hematite have been found at Maastricht- Belvédère Site C, well-dated to minimally MIS 7 by TL and ESR . Eight containers made of stalagmite fragments with traces of ochre and scraping marks have been found in two layers of Cioarei Cave in Romania, dated to MIS 3

Thus two sites (Cueva Antón, Fumane) document transport and coloring of exotic objects and their possible use as pendants, while the French sites, Ciaorei Cave and Maastricht- Belvédère site C show that use of red ochre was a component of Neandertal behavior, since at least 200-250 ka.

Extraction of large feathers from raptors, corvids and pigeons is documented by cut marks on distal wing and foot bones (elements that have no food value) in Mousterian layers from the site of Fumane (northern Italy and Gorham and Vanguard Caves (Gibraltar . Stronger evidence of the systematic use of bird elements is provided by cut marks on terminal phalanges of eagles at five sites in France and Italy (Fumane, Pech de l’Azé 1, Combe Grenal, Les Fieux, Pech de l’Azé IV) indicating the removal of the claw. Photos are available in the publications of first four sites .

The use of raptor claws is supported by the similar find of a terminal phalange of a raptor (probably a vulture) with a deep notch, interpreted as a pendant, from the cave site of Üçağizli (Turkey). The Initial Upper Paleolithic (cf. Emiran) and the Ahmarian layers at the site have yielded a large assemblage of ornaments, mostly pendants made from marine and freshwater mollusk shells. The raptor claw, the only non-shell ornament, comes from layer B (Ahmarian) dated to c. 41 ka [Fig 17 in . The find of Üçağizli in an Upper Paleolithic context provides support for the hypothesis that raptor claws could have been used as ornaments by Neandertals. Raptor claws and wing elements are overrepresented at two Epipaleolithic sites in Israel, Meged and Ohalo II and in both cases an interpretation of use of claws and feathers as ornaments was suggested .

*Social interaction between Neandertals and modern humans.*

As noted in the text, there are components of Neandertal cultural behavior that were demonstrably developed independently, such as the use of red ochre and manganese, personal ornaments like eagle claws, and bladelet production. In a recent paper it has been suggested that late Middle Paleolithic *lissoirs*, a type of specialized leather working bone tool, may constitute evidence for cultural diffusion from Neandertals to modern humans. One *lissoir* comes from layer 4 at Pech de l’Azé I dated to 51.4 ± 2.0 ka, thus predating the use of similar objects by AMH in Europe (see below “Lissoirs”). In the case of the Dufour bladelets in the Châtelperronian of Quinçay, where intrusions from overlying Aurignacian must be excluded by the absence of such levels at the site, a stimulus diffusion from the Proto-Aurignacian to Neandertals has been suggested .

It is interesting that data from Quinçay suggest that diffusion from Neandertals to modern human groups is also conceivable for another kind of artifact. In the same Châtelperronian levels of Quinçay there are six perforated animal teeth of different species (fox, wolf and cervids, see ref for photos of two). A similar conclusion was reached by Zilhao on the basis of the Arcy Grotte du Renne ornaments. Twenty eight grooved or perforated animal teeth occur in the Châtelperronian levels [X-VIII) of Grotte du Renne . Three perforated teeth (possibly of a bear) have been reported from Bacho Kiro (Bulgaria) in level 11 (the type assemblage of the Bachokirian ). The Bachokirian is considered broadly coeval with the Châtelperronian but its makers remain unidentified.

Perforated or grooved animal teeth occur in the Proto-Aurignacian but they are not common. Only one (a bear tooth) has been found in the Proto-Aurignacian level VII of Grotte du Renne 9: figs. 157-158], one pierced red deer canine comes from the Rothschild rock shelter (SE France) and two pierced incisors of a herbivore from Isturitz . To our knowledge none have been reported from pre-Aurignacian levels in the Near East, including the Initial Upper Paleolithic and Ahmarian levels of Üçağizli cave (Turkey), which yielded a large number of marine shell ornaments and one grooved raptor claw [50]. As noted by Zilhao pendants of animal teeth are unknown in Africa and the Levant, they seem to be a European novelty. Thus if the dates for the Châtelperronian at 45 to 41ka cal BP are accepted and the Protoaurignacian is dated to 39.9-41.5 ka cal BP, one could conclude that pendants of animal teeth were a Châtelperronian (Neandertal) invention transmitted to the Protoaurignacian .

It is important to note that perforated or grooved animal teeth used as pendants are very common in the Aurignacian: 486 teeth are recorded from 37 sites in Europe, two of which also contain pierced human teeth: Les Rois and Grotte des Hyènes at Brassempouy . Of course, the duration of the Châtelperronian and of the Protoaurignacian is much shorter and their spatial distribution more limited than that of the Aurignacian. Still this seems again to be a case of a novelty that was episodically present in Neandertal assemblages and later became a stable component of cultural behavior.

In conclusion the archeological evidence shows that personal ornaments and manipulation of ochre and manganese existed in the Neandertal world, well before the Châtelperronian.

**2. Neandertals had limited capacity for innovations**

***Hypothesis.*** This observation was first explicitly formulated by Mellars , who argued that rates of change in technology and regional variability were much more rapid in the Upper than in the Middle Paleolithic. Since technological change is slow throughout most of prehistory, increases in rates of technological change are thought to signal the emergence of abilities to develop new techniques and/or devices to cope with adaptive problems. This capacity is widely viewed as symptomatic of “modern” behavior and possibly signaling the emergence of a highly structured language . Klein also emphasized rapid increase in the rate of artifactual change through time and in the degree of artifact diversity through space in the Upper Paleolithic. This point of view was readily adopted by other scholars: Wynn and Coolidge for instance state that “Neandertals lacked the inventiveness, characteristic of people today”.

***Supporting evidence****.* The Upper Paleolithic technocomplexes of Western Europe, each characterized by distinctive “type fossil” forms both in stone and bone or antler implements, are confined to certain geographical areas and time periods. Each seems to last less than 10,000 years. The two main technocomplexes of the South African late MSA, the Still Bay (SB) and the Howiesons Poort (HP), considered to be two very dynamic and innovative phases with a large geographical distribution, were until recently thought to last less than 10,000 years each. The SB is dated at Blombos from about 75.5 to 67.8 ka and to about 70 ka at Sibudu . The HP at Klasies River, Sibudu and Rose Cottage has dates from 65 to about 60 ka .

***Refuting evidence*.** Recent research at the site of Diepkloof (Western Cape, South Africa) has shown that these two MSA technocomplexes have a significantly longer duration. In particular, the HP has now been subdivided in three phases: Early, dated by OSL to 109±10 ka and by TL to 105±10 ka; Intermediate, between 85±9 ka and 65±8 ka; and the Final HP (corresponding in time to and resembling the occurrences of Sibudu, Rose Cottage and Klasies, hence called the “classic” HP) dated to 52±5 ka . The SB is dated to 109 ±10 ka. The implications seemed clear: the two technocomplexes have a much longer duration than previously envisaged and they are not homogeneous in space.

However the OSL method used by Jacobs at the MIS4 - MIS3 sites has been criticized and the dates are judged unreliable . Moreover the nature of the Diepkloof SB and its relationship to the much younger assemblages of Blombos, Sibudu and Hollow Rock Shelter remain to be explored. The same is true for the HP assemblages dated to MIS 5 at Diepkloof. In contrast the dates for the Post-HP technocomplex based on several dating methods (TL, OSL, ESR, ^14^C, U-series) are more secure and can be compared to the dates of Upper Pleistocene technocomplexes in Europe, as in Table 1 (text).

Middle Paleolithic industries in Europe dated between MIS 5 to MIS 3 show clear spatio-temporal distributions . The Mousterian of Acheulian Tradition of Southwest France dates to between 70 and 40 ka, based on dates obtained from six sites, and hence lasted approximately 30,000 years. The Quina Mousterian dates to between 73 and 40 ka, based on dates obtained from six sites . The *Keilmessergruppen* (KMG) in Central Europe have bifacial tools which are technologically and typologically well-defined guide fossils. The KMG can be subdivided into separate inventory types in a clear chronological succession and with regional differentiation .

In conclusion, the pace of change in the last phases of the Middle Paleolithic, prior to the arrival of modern humans, is comparable to that of the late MSA in South Africa.

**3. Neandertals were less effective hunters**

The idea that scavenging was an important pattern of subsistence behavior of early hominins and the Neandertals was first suggested by Binford . Later Stiner argued that Neandertals in Central Italy had a flexible adaptation alternating scavenging with hunting, which became prevalent only in late Neandertal times. These ideas have been disproven by a number of studies , and accumulated evidence shows that hunting was the main method of meat procurement by Neandertals . The scavenging hypothesis is dead and we will not discuss it any further. However the idea that Neandertals were less effective hunters has been reformulated in terms of their hunting methods and weapon technology.

**Hunting methods**

***Hypothesis***. Neandertals hunted in small groups in long dogged pursuits across the landscape, targeting the largest prey animals. Their killing ability was limited to thrusting spears equipped with simple stone points . Their encounter technique involved high risk close contact with large prey animals resulting in high trauma rates; patterns and frequencies of Neandertal bone breakage fall outside the norm for modern humans , except for twentieth-century rodeo riders . In contrast to modern humans, Neandertals did not focus on communal hunts and herd intercept at times of migration and did not make efficient use of the landscape to hunt their prey. Their hunting efficiency, as measured by time and effort expended to kill their prey was, less than that of modern humans in Africa and in Europe .

***Supporting evidence* *for organizational techniques and killing abilities.*** Neandertal skeletons show extreme physical activity and healed upper-body fractures indicating close combat with spear thrusting. Their sites were small, focused around a single hearth with little structured use of space . The implication is that the Neandertal small sites do not indicate large group organization of the type typical for groups with communal hunts.

***Refuting evidenc***e.

*Traumas.* This reading of trauma in Neandertals versus modern humans has now been retracted by Trinkaus himself , stating that “assessment of Late Pleistocene modern humans indicates a similar pattern to that of Neandertals…explaining the trauma pattern solely as a result of Middle Paleolithic hunting weaponry or conversely using the Neandertal trauma pattern to argue for ineffective Middle Paleolithic weaponry, should be abandoned”.

*Communal hunts and tactical thinking*. A number of sites dated between MIS 9 and MIS 3 show evidence indicative of organized hunts, using topographic setting for killing one or two species of large mammals (Table S1). Four sites show the use of cliff faces and rocky barriers associated with karstic systems for bringing down large mammals. At Schöningen the remains are in organic muds and the age distribution indicates the presence of at least one family group of horses. The fossil material is well preserved with little variation in weathering suggesting rapid accumulation. The French sites appear to have been formed by a number of different hunting episodes, with the MIS 6 site Coudoulous containing the remains of 232 bison. The evidence shows that Neandertals were able to organize game drives using landscape features as natural traps, intercepting groups of animals at repeatedly used locations. At other times large and medium size mammals were hunted individually. Scheduled hunting is not, as suggested by some , the exclusive domain of modern humans.

Table S1. Sites with evidence of repeated kill episodes using topographic settings. Data from and references therein.

| **Site** | **Age** | **Main fauna** | **MNI** | **Topographic setting** |
| --- | --- | --- | --- | --- |
| Schöningen  13 II-4  (Germany) | MIS 9 | *Equus mosbachensis* | 20-25 | Muddy shore of a lake |
| La Cotte St. Brelade, (Jersey) layers 3 and 6. | MIS 6 | *Mammuthus primigenius, Coelodonta antiquitatis* | 7, 11  2, 3 | Base of a deep ravine |
| Coudoulous (SW France)  Layer 4 | MIS 6 | *Bison priscus* | 232 | Karstic depression, open air |
| La Borde (SW France) single layer | MIS 7 or 5 | *Bos primigenius* | 40 | Karstic depression, open air |
| Mauran (SW France) | MIS 3 | *Bison priscus* | 137 | Base of an escarpment with rocky barrier |

*Internal site structure* *and group size*. Spatially organized activities and well delimited activity areas have been documented by refitting at a number of Middle Paleolithic open-air sites, such as Maastricht-Belvédère site K (Netherlands, MIS 7) , La Folie, SW France, estimated age MIS 5e , Ksiecia Jozefa layer III (MIS 3) and at rock shelters such as Abric Romani (Spain, MIS 3) and Tor Faraj (Jordan, 55 ka) . Intra-site spatial analysis combined with refitting and hearth distributions documents two living floors at Tor Faraj, with an estimated range of 15 to 20 persons as hearth-side occupants in each living floor, an area of 136 m^2^ and multiple, spatially segregated task-specific areas. These estimates correspond to the number of people commonly seen in modern foraging bands.

Evidence of complex internal site structure is also indicated at Kebara , at the open air site of Quneitra [90] and at Abric Romani . At the latter site the spatial distribution of wood remains in different areas of the shelter has been interpreted as revealing systematic collecting and even storing of fuel sources (twigs and small branches for lighting, large branches and trunks) to supply the 37 hearths of layer M, dated to ca. 50 ka.

*Hunting efficiency.* It may be worth noting that hunting efficiency is hardly a measure of modernity. The descriptions of San hunting techniques and recovery rates indicate clearly that they invest an inordinate amount of time tracking wounded animals across the landscape (because their bows are weak and they use a slow-acting poison) and their recovery rates are relatively low, because they often lose their animals to other predators. Return rates are also low for the Hadza, who get higher caloric returns from gathering .

**4.Neandertal weaponry was inferior to AMH projectile technology**

The hunting weapons of Neandertals involve a number of arguments based on anatomical and archeological evidence.

**4.1 *Hypothesis.*** Neandertals only had thrusting spears

***Supporting evidence***. Patterns of bone frequencies and breakage (see above “Traumas”) and the upper limb morphology of Neandertals indicate close combat with spear thrusting. Upper limb morphology associated with projectile throwing occurs in Upper Paleolithic humans but is absent in Neandertals .

***Refuting evidence****.* In contrast to the Lehringen spear, interpreted as a thrusting spear, experiments and measurements show that the Schöningen spears had ballistic qualities indicating that they were thrown as javelins (and refs therein). The humerus and shoulder morphology associated with overarm throwing were already present in *Homo erectus* . The particular characteristics of Neandertal humeri may reflect adaptation to regular scraping activities and not spear thrusting .

**4.2 *Hypothesis.*** Complex projectile technology gave early modern humans a competitive advantage over Neandertals*.*

Modern humans used spear throwers and/or bow and arrow to launch their projectiles. This would have given them an advantage in their competition with Neandertals .

***Supporting evidence***. Of the variety of measurements proposed to identify prehistoric stone points as projectile points, the tip cross-sectional area (TCSA) is a widely applied one . Based on a series of North American arrowhead and spear-thrower launched dart tips as well as on experimental studies, Shea has suggested that early stone projectile points should not present higher TCSA values than the more recent ones in his sample. TCSA values of a series of Middle Paleolithic Levallois points were significantly larger than the ones from his control sample, whereas the values from EUP points did not differ from the control sample points. Values of TCSA from Blombos and other South African sites also suggested to him that some of the points were used as spear-thrower darts . These results were interpreted as implying that Neandertals did not have a complex projectile technology, in contrast to late MSA and EUP humans.

***Refuting evidence***. The TCSA values from Blombos used by Shea are incorrect, because he used a data base which contained preforms and unfinished pieces (thus obtaining small values from broken pieces) and because he excised large values using an arbitrary cut-off point .

A recent study of Australian hafted dart projectile points shows that TCSA values of Australian macro-blade darts are significantly larger than the ones from the North American dart points; in fact, their mean is significantly larger than the values Shea obtained for (early) Middle Paleolithic Levallois points (compare Fig 4 in ref with Fig. 6 of ref. ). In other words the TCSA statistics have a limited value and cannot be seen as documenting a particular delivery system.

For instance, Mousterian points from Italian and Spanish sites have significantly lower TCSA values than those used by Shea for thrusting spears and are comparable to those for darts . We are not suggesting that Neandertals had spear-throwers. In fact, the key issue illustrated by the Australian dart points is the importance of variability, both within prehistoric and extant human hunter-gatherer populations . Interestingly, hunter-gatherers in Australia are not known to have used bow-and-arrow. Tasmanian aboriginals did not even use spear throwers, and only had hand delivered spears .

**4.3 *Hypothesis.*** AMH had bow and arrow, Neandertals only hand-cast spears

This idea is based on two separate claims: a) small quartz pieces used to tip arrows occur in the Howiesons Poort (HP), and b) backed bladelets made of heat-treated silcrete and used as arrow-heads represent a weapon technology originating 71,000 years ago in a pre-HP industry in South Africa.

*a) Quartz-tipped arrows in the Howiesons Poort*

***Supporting evidence***. A hypothesis of quartz-tipped arrows in the HP of South Africa, first proposed in 1982 for the Klasies River Main Site has been suggested for the Sibudu HP by Wadley and Mohapi and supported by Lombard based on the presence of very small quartz segments (<2.5 cm) interpreted as transverse arrowheads. They conclude that projectile technology was developed in the MSA of South Africa before 60 ka.

***Refuting evidence***. This claim is poorly supported owing to (i) the very small number of possible arrowheads compared with the total of backed pieces at both sites, cf. below, (ii) the fact that equally small or smaller pieces were used as barbs on spears in the Upper Paleolithic, and (iii) the total lack of evidence for arrowheads in post-HP assemblages of the following 15-20,000 years, including Sibudu . Bow and arrow are generally recognized as a successful weapon, widely adopted on most continents, used for hunting a wide range of game in forested as well as in grassland settings. The apparent subsequent loss of this successful technology is explained as a historical contingency , that is, a chance historical event, due to undetermined or random factors. A simpler alternative might be that there was no bow and arrow technology to loose.

It is only at about 44-42 ka that there is good evidence for the use of (poisoned bone) arrows at Border Cave in South Africa , well after the hypothesized migrations of modern humans into Eurasia . At the White Paintings rock shelter in Botswana bone points that appear to have been parts of reversible arrowheads are dated to 37-35 ka .

As indicated above*,* the number of quartz pieces is very small. At Klasies there are 14 pieces < 2.5 cm of quartz and silcrete of a total of 109 backed pieces of all raw materials in layer 20 . At Sibudu the total number of quartz backed pieces from the HP layers interpreted as arrowheads is 14 of a total of 281 backed pieces of all raw materials .

Impact notching on thin edges has been observed in experiments with replicated backed pieces hafted transversely and used to support an interpretation of transverse arrowheads for 9 small quartz segments from HP layers of Sibudu . However the impact notches so described do not have a negative bulb and cannot be distinguished from micro-fractures and pseudo-retouches produced by trampling or post-depositional damage in the sediments . We do not consider this kind of notching diagnostic of impact.

Experimental studies using 265 replicated Kebaran and Natufian microliths hafted on arrows and shot with a bow report bending fractures (type b3 in fig. 3 and Table 2 in and step-terminating scars (type b3 and d2m in figure 3 and Tables 2-3) for transversely hafted microliths. The scars are unlike the notches described in Lombard and Pargeter . The latter type (d2m) also occurs on microliths used as barbs. For this and other reasons, especially the absence of evidence for bow and arrow technology in post-HP times , we consider the hypothesis of quartz-tipped arrows in the HP of Sibudu

poorly supported. Even if one should consider the bow and arrow hypothesis a possibility, the record shows it was a short-lived experiment.

b) *Backed bladelets used as arrow tips represent a weapon technology originating 71,000 years ago in South Africa.*

***Supporting evidence****.* Small backed bladelets, made on heat-treated silcrete, occur at Pinnacle Point Site 5-6 and are found in layers dated between 71 and 60 ka . The Pinnacle Point evidence supposedly indicates that the production of backed bladelets is even earlier than the HP and represents a long-lasting technology that “conferred substantive advantage on modern humans as they left Africa and encountered Neandertals equipped with only hand-cast spears” .

***Refuting evidence***. The proposed date of 71 ka for the origin of backed bladelet technology has been shown to be incorrect since backed bladelets occur much earlier at Diepkloof in the Early HP, dated by OSL to 109±10 ka and by TL to 105±10 ka . We will not deal with this chronological problem here, only with the claim of a technological supremacy of early moderns.

The idea that backed bladelets provided modern humans with projectile weaponry that gave them a huge advantage on Neandertals, once modern humans moved out of Africa is difficult to prove with the existing data. Intentional bladelet production appears at Umm-el Tlel (Syria) at about 40,000 BP (based on uncalibrated ^14^C dates) in the Early Ahmarian at about 41.6 - 40.3 ka cal BP at Ksar Akil and Üçağikli ] and in the Proto-Aurignacian at c. 41.5-39.9 ka cal BP . The Emiran or Initial Upper Paleolithic industry (IUP) of Boker Tachtit (Israel) levels 1 and 2, dated to about 47,280 ± 9050 and 46,930 ± 2400 (these are dates for Level 1 but note the very large standard deviation) lacks backed bladelets , while the similar IUP assemblages of Tor Sadaf A and B (Jordan) have only a very small number of retouched bladelets . In Emiran, Bohunician or Bachokirian industries points predominate and were probably used to arm spears . The success of bladelet production begins with the Ahmarian in the Levant and in the Proto-Aurignacian in Europe. According to recent OSL dates, microblade technology appeared in India at 44 ± 2 ka (weighted average of four dates) at the open-air site of Mehtakeri roughly comparable in age to similar dates for the Ahmarian.

If the age of migration of modern humans into Southwest Asia and India is set prior to 60 ka, there is a difference of more than 15,000 years between the hypothesized migration and the occurrence of bladelet production in the Near East and India.

If the migration is set at c. 60-50 ka this would rule out South African populations, since by 60 ka the HP tradition of backed tools made on blades and bladelets produced by soft stone hammer had given way to the post-HP assemblages characterized by a variety of flake tools and blades produced by hard hammer percussion, but without backed blades .

If modern humans migrated from East Africa shortly after 100 ka the idea of an intergenerational transmission of a long-lasting technology of backed pieces helping the spread of humans out of Africa is again ruled out by the difference of c 60,000 years between the hypothesized migration, the Neandertal demise and the systematic production of bladelets in the Near East and India. The latest Neandertal currently known from the Levant is the adult male skeleton from Amud Cave (Israel) with an ESR date of 53 ± 8 ka on tooth enamel corresponding well with TL dates on heated flints from the same deposits .

Finally, if the new technology spread from Africa to the rest of the world accompanying the dispersal and supremacy of modern humans, why is it that the hunting arsenal of Australian Aboriginals does not include bow and arrow? In North America the spear-thrower was probably introduced by the first settlers but the advent of the bow is much later, roughly around 2300-1300 BP in western North America, even later in the eastern part of the country .

In conclusion, the idea that bow and arrow technology was a factor in the demise of Neandertals remains as yet unsupported.

**5. Neandertals had a narrow diet that proved unsuccessful in competition with modern humans that had a more diversified diet.**

***Hypothesis****.* Neandertal diet was restricted to large and medium size herbivores. Early modern humans had a broader diet, including significant quantities of aquatic foods. Neandertals were unable to acquire small fast game (birds, wild rabbits) and did not exploit plant resources .

***Supporting evidence***. Carbon and nitrogen isotope values of Neandertal bones from northern regions or cold environments such as Belgium, Germany, Southwest France, and Croatia, indicate that Neandertal were top-level carnivores obtaining most of their proteins from large herbivores such as bovids and horses . More recently isotope analysis has been applied to Neandertal remains from 2 Spanish sites, Cova Negra and El Salt. The results also show that Neandertals consumed large amounts of meat .

***Refuting evidence***. Isotope analyses have a low resolution detecting plant protein when the diet is dominated by animal protein. Nutritional ecology studies have stressed that Neandertals would have needed more than just large mammal protein, including a range of micronutrients not readily available through terrestrial mammals . A broader picture is provided by zooarchaeology and studies of plant remains.

The faunal remains in Mediterranean regions indicate an abundance of small ungulates i.e. smaller than red deer (gazelle, roe deer, fallow deer, caprids) at cave sites such as Kebara, Misliya, Amud and Qesem . Small prey, such as wild rabbits, is well documented by cutmarks and anthropic fractures at Bolomor Cave (Spain) in layers XVIIc (dated to MIS 9, NISP = 457) layer XI (MIS 6, NISP = 262) and layer IV (MIS 5e, NISP = 789) , which also yielded abundant evidence for tortoise consumption at Les Canalettes layer 4 (France, c 70 ka; NISP = 1209) and at Abri du Maras (Ardèche Valley, France, beginning of MIS 4, only a few remains but with two tools also showing leporid hair fragments on their working edges .

Layer XI of Bolomor Cave has also yielded 202 NISP of the genus *Aythya* (diving duck) corresponding to a MNI of 8. This species which lives in marshes and lakes is represented by various elements, especially leg bones and the coracoids. Cutmarks and bone breakage indicate human consumption . At the late Middle Paleolithic open air site Salzgitter-Lebenstedt (Germany), cut-marked bones of duck and swan were identified . The capture of raptors, corvids and wood pigeons for removal of wing feathers is documented by cutmarks and bone breakage at Fumane in northern Italy and at Gorham and Vanguard Caves in Gibraltar . At the latter sites the capture of birds may perhaps have been related to use of feathers as ornaments (see *Hypothesis 1*). The capture of hare, beaver, marmot and some carnivore (bear, fox and mustelids, documented by cutmarks and possibly related to the use of pelts) is documented again at Fumane; cutmarks also occur on beaver bones at Grotta Maggiore di San Bernardino in northern Italy . Cutmarks and bone breakage patterns document the capture of rabbits and birds at an even older site in Spain, Gran Dolina level TD 10-1, TL dated to MIS 9 .

Evidence of plant consumption by Neandertals is provided in Table S2.

Table S2. Plant consumption by Neandertals*.* Data based on

| **Site** | **Archeological evidence** | **Kind of food** |
| --- | --- | --- |
| Kebara (Israel) | 4205 charred seeds and fruit, phytoliths | Legumes (wild peas), acorns, pistachios |
| Amud (Israel) | Phytolith analysis | Grass seeds |
| Gorham’s Cave  (Gibraltar) | Charred nuts | Pine nuts |
| Spy (Belgium) | Microfossils in dental calculus | Water lilies? grass seeds |
| Shanidar (Iraq) | Microfossils in dental calculus | Date palms, grass seeds |
| El Sidrón (Spain) | Microfossils in dental calculus | Cooked carbohydrates |
| Sima de las Palomas (Spain) | Microfossils in dental calculus | Grass seeds and other plant types |

*Fishing.* Remains of freshwater fish (trout, chub, eel, NISP =21) are documented in various Mousterian levels of Castelcivita Cave (Italy) older than 40 ka . The Mousterian layers there underlie the Uluzzian, Protoaurignacian and the Campanian ignimbrite ashes. Additional evidence of freshwater fish consumption is provided by the sites of Payre in France , Kudaro in the Caucasus ] and Abri du Maras (MIS 4; mostly cyprinids and percid, NISP = 167) [[14](#_ENREF_141)1]. At Abri du Maras the contribution of non-human predators to the accumulation of fish bones and scales is excluded by the absence of any signs of chewing and digestion. More taphonomic studies are needed to ascertain the role of fishing activities in the diet of Neandertals. It needs to be stressed that in Europe high numbers of fish remains are documented only in the late Upper Paleolithic [132].

In conclusion, recent research by different scholars using different analytical methods shows that Neandertal diet was more diverse than generally acknowledged, and varied according to different environments. A broad-based subsistence was already in place by the second half of the Middle Pleistocene and was not limited to Mediterranean regions, as indicated by sites such as Spy, Kudaro, Shanidar, El Sidrón, Payre and Abri du Maras, going from Belgium to Iraq and from the Atlantic sea shore to the Caucasus.

**6. The use of traps and snares to capture animals is the exclusive domain of modern humans.**

***Hypothesis***. It is often suggested that the capture of fast game such as birds and wild rabbits implies the use of traps and snares. For some authors [80] the use of snares and traps is an indication of advanced planning abilities and complex cognition which are attributes of the modern mind and hence relatively recent developments. Use of plant fibers for binding, weaving, basketry and possibly net-making is documented in the Upper Paleolithic of Dzudzuana Cave in Georgia at 30 ka and at the roughly contemporary Gravettian sites of Dolní Vestonice and Pavlov 1 in the Czech Republic . High numbers of small fur-bearers like hares and foxes have been recovered from the Gravettian sites.

***Supporting evidence***. The supposedly rare presence of bird and lagomorphs remains in the Middle Paleolithic has been interpreted as due to absence of trap-related technology and related to low population densities . Indirect evidence of using traps in the MSA has been argued by Wadley based on the abundance of tiny blue duiker remains, easily captured with nets or snares, in the Sibudu Howiesons Poort (c. 65-61 ka) faunas.

***Refuting evidence***. Our data on the capture of small fast game in the Middle Paleolithic (*Hypothesis 5*) suggest that catching smaller and fast moving game, thought to indicate the “enhancement of working memory capacity” [80], was already within the realm of Neandertals, as early as Middle Pleistocene times. This is supported by the presence of twisted fibers (found as residue on 12 stone artifacts) at Abri du Maras (France, early MIS 4]. Since fibers are not twisted in their natural state they provide tantalizing evidence for the manufacture of strings or cordage which would facilitate the manufacture of multi-component technology such as snares .

**7. Modern humans had larger social networks**

***Hypothesis***. Long-distance exchange networks can operate in times of scarcity to allow access to distant areas where resources are plentiful, thus acting as a buffer against environmental downturns and increasing the long term survival of groups . Modern humans established such far-reaching extended social networks, unlike those of the Neandertals .

***Supporting evidence.*** Long distance transfers of lithics and especially shells, inferred to reflect the geographical size of social networks, do increase in the Upper Paleolithic of Europe, both in terms of quantity of transported (lithics) items and the distances from their sources, though the changes are not dramatic. A number of Upper Paleolithic assemblages (n=23) documents maximum distances covered of > 300 km, from the Aurignacian onward, with the largest distances documented for the Gravettian and the Magdalenian, virtually all entailing movement of shells .

At Ortvale Klde (Georgia) the source of high-quality obsidian was about 100 km to the southeast. A comparison of the late Middle Paleolithic and the early Upper Paleolithic assemblages shows higher proportions of obsidian in the early Upper Paleolithic. In the early Upper Paleolithic obsidian is represented by full reduction sequences while in the late Middle Paleolithic obsidian artifacts appear as small debitage and heavily reduced tools. The conclusion is that Neandertals moved only infrequently between regions and their social relations were structured into smaller territories than those of the Upper Paleolithic modern humans .

However all these are comparisons between Neandertals and their younger successors. We should compare Neandertals with their modern human contemporaries in Africa and this is the evidence we consider here. McBrearty and Brooks report distances of transport of obsidian at MSA sites in East Africa up to 240 km at the Nasera Rock Shelter and 320 km in Bed VI at Mumba (both sites in northern Tanzania). In the MSA of South Africa fine-grained raw materials have been reported as non-local. Deacon and Wurz suggest that the backed pieces of the HP had a symbolic value and may have been used in a reciprocal exchange network.

***Refuting evidence***. The quantities of obsidian transport in the early MSA of East Africa are small, indicating weak group interactions . This is also true of Middle Paleolithic assemblages where maximum transport distances in Western Europe are around 110-120 km (contrary to the opinion of ).

It is only at the end of the MSA or during the MSA/LSA transition (older than 40 ka) in assemblages like Prolonged Drift and Ntumot in Kenya, that the proportions of obsidian from sources 45-70 km away increase, possibly indicating more intensive intergroup exchange . In the later Middle Paleolithic record of Central and Eastern Europe maximum distances are much larger than in Western Europe (> 200 km), usually interpreted as a result of ecological differences: in exceptional cases trans-Carpathian transport moved materials up to 280 km . While these large distances again mostly relate to isolated and heavily reduced pieces, the Last Interglacial (MIS5) levels at Kulna cave in the Czech Republic , yielded exotic materials in the form of blanks and rough cores, imported over a distance of 230 km , i.e. comparable to the form of transport recently described for the 50-100 ka old deposits from the White Painting Shelter site in Botswana .

The conventional view that HP was associated with long distances of raw material transport is incorrect. At several sites raw materials were local or transported over short distances. At Rose Cottage the main raw material (frequencies of cores and retouched pieces > 90%) is opaline which occurs in the Caledon river gravels 8-10 km from the site . At Sibudu the main raw materials are hornfels, dolerite, quartz and quartzite, all of which occur near the site or at a distance of about 15 km . At Klasies River quartz was interpreted as non-local by Wymer but it is very likely local since it occurs as seams and cobble inclusions within the Table Mountain Sandstone, the parent material of the Klasies River Cave. The sources of silcrete are not known; however the presence of rolled cortex on several silcrete cores and retouched pieces suggests beaches (which are adjacent to the site) or river gravels as one of the sources . At Blombos there seem to have been two sources of silcrete: from deposits 30 km north of Blombos and from river valleys which exit into the ocean 20 km east and west of the site . At Diepkloof high-quality silcrete comes from sources > 20 km from the site . At Border Cave the main raw material in the HP and younger periods is rhyolite which is the local bedrock. Another, less abundant, raw material is chalcedony, which occur as vesicles in the rhyolite and these seem to be most common 40 km from the site . In sum, the MSA and Middle Paleolithic records for raw material transfer distances are not significantly different.

Finally the idea that Neandertals groups were “limiting themselves to a single river valley and only occasionally did they venture farther afield” is refuted by abundant evidence for transport of raw materials across major river valleys in the Middle Paleolithic of western and central Europe 2].

**8. Modern human populations were larger than Neandertal populations**

***Hypothesis***. Modern human initial populations entering Neandertal territory were significantly larger, in southwestern France an estimated ten times, than the regional Neandertal populations: “…numerical supremacy alone must have been a powerful, if not overwhelming, factor in direct demographic and territorial competition between modern humans and Neandertals” .

***Supporting evidence***. Mellars and French have attempted to estimate population numbers for late Neandertals (as reflected by Mousterian of Acheulian Tradition and Châtelperronian sites) and the earliest modern humans (thought to be reflected in Aurignacian sites) for southwestern France using archeological proxies: (i) data on total numbers of occupied sites in the study region during the Neandertal–to–modern human transition period, (ii) the overall intensity of occupation in these sites, as reflected by the accumulation rates of stone artifacts and animal bones in these sites and (iii) data on the overall spatial extent of the archeological occupation levels, as a potential reflection of the sizes of the human groups who occupied the sites in the period at stake. With the arrival of Aurignacian producing groups they observed a sharp increase in the total number of occupied sites, much higher densities of occupation and larger areas of occupation in the sites, thought to be proportional to the number of people occupying a site. They conclude that earliest modern human populations penetrated the region in at least ten times larger numbers than those of the local Neanderthal populations in the same regions.

***Refuting evidence***. The Mellars and French population size hypothesis has recently been reviewed *in extenso* by Dogandžić and McPherron , who analyzed each of the three inferred proxies for population sizes, in terms of their assumptions, their significance and their archeological visibility. Estimating population numbers using archeological proxies is a challenging enterprise, as acknowledged by Mellars and French themselves. But some of the problems were created by their own methods. For instance, the list of sites inhabited by the last Neandertals does not include sites belonging to different technocomplexes (Denticulate Mousterian and Quina Mousterian) and yet falling into their selected time span (even more sites can be added to those listed by Dogandžić and McPherron). Stone artifacts and faunal densities are generally calculated by cubic meters of excavated deposits. Mellars and French instead calculated densities by square meters per 1,000 years of estimated duration of each technocomplex, a debatable procedure since it assumes that a local occurrence of a technocomplex spans the entire known chronological range of that technocomplex. The proxy used to calculate the number of people living at a site is also affected by various factors, such as a) the small size of some caves which constrains the use of space, b) in the case of old excavations by inadequate documentation of the site size, and c) changes in site configuration through erosion. Dogandžić and McPherron show in detail that the Mellars and French analysis is severely flawed, concluding that “…Mellars and French have biased their results in favor of a conclusion that AMH populations were larger than late Neandertal populations. With a more conservative set of assumptions and expected outcomes, their own data show roughly equivalent values for the respective proxies meaning that they have not demonstrated a tenfold increase in Western Europe at the Neandertal-to-modern human transition”(p. 313). The Mellars and French rebuttal fails to address their critique adequately, and in fact even underlines it, by admitting that taking into consideration “…the maximum allowance for factors of this kind…” (as discussed by Dogandžić and McPherron) would lead “..to a more modest increase in our calculated demographic estimates - say down to a five-fold as opposed to ten-fold population increase “.

Conard uses a better controlled set of variables (size of excavation area and number of lithic and organic artifacts by cubic meter) to reach an approximation of occupational intensity and mobility of Neandertals versus Aurignacian groups in the Swabian Jura in southern Germany, a hilly region rich in cave sites with Middle Paleolithic and Aurignacian deposits. The study area is approximately 5000 km^2^, much smaller than the study area of Mellars and French (75,000 km^2^). He concludes that Neandertal population densities in the region appear to have been uniformly low, lower than in the subsequent Aurignacian period. There are however significant gaps and uncertainties in our knowledge, as noted by the author himself. Open-air sites had to be excluded from analysis due to lack of preservation of organic artifacts, so only ten multi-component cave sites could be analyzed The high residential mobility typical of Neandertals probably resulted in brief periods of occupation and low densities of artifacts. In sum, archeological data suggest -but do not provide decisive evidence- that small population size may have played a role in the replacement of Neandertals by AMH in that region.

**9. Hafting, heat treatment and modern cognition**

Complex hafting procedures which required use of fire and the use of fire for pitch production are discussed in the text. In fact, heat treatment was a component of Neandertal technology. Neandertals used fire to heat-treat existing natural materials, such as bitumen for hafting purposes. This is clear from 70,000-y-old tools with traces of bitumen on their surfaces from the site Umm El Tlel (Syria). The bitumen was collected at 40 km from the site . Bitumen was also recovered from a Mousterian tool at the site of Gura Cheii-Râşnov Cave in Romania .

The idea that Neandertals had unsubstantial hearths, less effective than those of modern humans in protecting from the cold tends to persist but there is ample published evidence that it is incorrect .

**10. Climate as a factor in the extinction of Neandertals**

*Hypothesis*. The Campanian Ignimbrite (CI), the largest volcanic eruption in Europe, from the Phlegrean Fields north of Naples, spread ashes over an area of 5,000,000 km^2^, eastward to Greece, Bulgaria and into Russia. As it occurred right after the onset of the Heinrich Event 4, dated to c. 40 ka, and characterized by very cold and dry climate, it is likely to have caused a volcanic winter .

The climatic deterioration and resulting population redistribution may have triggered the major cultural changes of the Middle to Upper Paleolithic transition, ultimately leading to the decline of Neandertals .

*Supporting evidence*. The chemical composition, age and spatial distribution of the CI are well documented. At some sites (Crvena Stijena in Montenegro, Franchthi Cave in Greece and Temnata (Bulgaria) the tephra occurs above the last Mousterian layer and below the Upper Paleolithic . However, no strong evidence in support of the idea of a demographic crisis of Neandertals is provided by Fedele et al. . Giaccio et al. consider the climatic instability of MIS as a potential factor involved in the cultural changes of the Middle to Upper Paleolithic transition.

*Refuting evidence*. The discovery of cryptotephra ash deposits (layers not visible to the naked eye) above the Uluzzian, Proto-Aurignacian, Initial Upper Paleolithic and Dabban layers at sites such Haua Fteah (Libya) and four sites in Central Europe (Klissoura, Golema Pesht, Kozarnika and Tabula Traiana) combined with previously known occurrences of the tephra above Proto-Aurignacian deposits (at Serino and Castelcivita, in Italy) and the Initial Upper Paleolithic (at Kostienki-Borschevo, Russia) show that neither Neandertals nor modern humans were adversely affected by the climatic cooling . We conclude that the decline of Neandertals was not associated with the Heinrich Event 4 and the CI eruption.

**11. The Toba eruption as an indirect factor in the extinction of Neandertals**

*Hypothesis.* The super-eruption of Mount Toba (Sumatra) at about 75 ka caused a “volcanic winter” of such a catastrophic scale that it led to a human genetic bottleneck, reducing the number of AMH in Africa to near extinction. The modern human population expanded again later and migrated out of Africa eventually causing the demise of Neandertals.

*Supporting evidence*. The bottleneck would explain the limited modern-day genetic diversity. Release from the bottleneck with a significant increase in population could have coincided with a climatic amelioration of the Dansgaard-Oeschger event 19. The chronology of the earliest LSA in East Africa at about 40-50 ka for the Nasampolai industry at Enkapune Ya Muto and the Middle to Upper Paleolithic transition fits the genetic evidence.

*Refuting evidence*. There is no associated evidence of mammal decline or extinction even in environmentally-sensitive species. There is no evidence of habitat reduction at that time in Africa . More important, analysis of crypto-tephra (ash) from the Toba eruption in a layer of Lake Malawi (East Africa) and accompanying sediments shows that there is no major change in temperature or sediment composition. The Toba eruption did not have a significant effect on the climate of East Africa and was not the cause of a human bottleneck in Africa at around 75 ka . Likewise, there is archeological evidence that the Toba eruption did not affect the behavior of populations inhabiting peninsular India .

It has been claimed that the response of modern humans to the unpredictable environment of the period was to extend inter-group cooperative networks to reduce risks while Neandertals continued to live in small territories with limited intergroup interaction, often involving violence and cannibalism. These differences may have been responsible for the replacement of Neandertals by African modern humans . Since there was no “volcanic winter” in Africa, such inferences must also be rejected.

**References**

1. Mellars P (1989) Major Issues in the Emergence of Modern Humans. Curr Anthropol 30: 349-385.

2. Demars PY (1992) Les colorants dans le Moustérien du Périgord. L’apport des fouilles de F. Bordes. Bull. Soc. Prehist. Française 47:185-194.

3. Mellars P (1996) The Neandertal Legacy: An Archaeological Perspective from Western Europe. Princeton University Press, New Jersey.

4. Soressi M, D'Errico F (2007) Pigments, gravures, parures : les comportements symboliques controversés des Néandertaliens. In: Vandermeersch B, Maureille B editors. Les Néandertaliens. Biologie et culture*s*. Editions du Comité des Travaux Historiques et Scientifiques, Paris, pp 297-309.

5. Klein RG (2009) Darwin and the recent African origin of modern humans. Proc Natl Acad Sci 106:16007-16009.

6. Klein R (2003) Whither the Neanderthals? Science 299:1525-1527.

7. Stringer CB, Gamble C (1993) In Search of the Neanderthals: Solving the Puzzle of Human Origins (Thames and Hudson, London/New York).

8. Mellars PA (2005) The Impossible Coincidence. A Single-Species Model for the Origins of Modern Human Behavior in Europe. Evol Anthropol 14:12-27.

9. Conard NJ (2010) Cultural modernity: Consensus or conundrum? Proc Natl Acad Sci 107:7621-7622.

10. d'Errico F, Villa P (1997) Holes and grooves: The contribution of microscopy and taphonomy to the problem of art origins. J Hum Evol 33:1-31.

11. d'Errico F*,* et al. (2003) Archaeological Evidence for the Emergence of Language, Symbolism, and Music - An Alternative Multidisciplinary Perspective. J World Prehistory 17:1-70.

12. d'Errico F, Henshilwood C, Vanhaeren M, van Niekerk K (2005) *Nassarius kraussianus* shell beads from Blombos Cave: evidence for symbolic behaviour in the Middle Stone Age. J Hum Evol 48: 3-24.

13. Henshilwood CS*,* et al*.* (2002) Emergence of Modern Human Behavior: Middle Stone Age Engravings from South Africa. Science 295:1278-1280.

14. Henshilwood CS, d'Errico F, Watts I (2009) Engraved ochres from the Middle Stone Age levels at Blombos Cave, South Africa. J Hum Evol 57: 27-47.

15. Brown KS*,* et al. (2009) Fire As an Engineering Tool of Early Modern Humans. Science 325: 859-862.

16. Botha R (2008) Prehistoric shell beads as a window on language evolution. Language and Communication 28:197-212.

17. Botha R (2010) On the Soundness of Inferring Modern Language from Symbolic Behaviour. Cambridge Archaeological Journal 20:345-356.

18. Balari S*,* et al. (2011) The archaeological record speaks: bridging anthropology and linguistics. Int J Evol Biol 2011(Article ID 382679):1-17.

19. Botha R (2009) Theoretical underpinnings of inferences about language evolution: the syntax used at Blombos Cave.In: Botha R, Knight C editors. The Cradle of Language. Oxford University Press, New York, pp 93-111.

20. Porraz G*,* et al. (2013) The MSA sequence of Diepkloof and the history of southern African Late Pleistocene populations. J Archaeol Sci 40:3542-3552.

21. Texier PJ*,* et al. (2010) A Howiesons Poort tradition of engraving ostrich eggshell containers dated to 60,000 years ago at Diepkloof Rock Shelter, South Africa. Proc Natl Acad Sci 107: 6180-6185.

22. D’Anastasio R*,* et al. (2013) Micro-Biomechanics of the Kebara 2 Hyoid and Its Implications for Speech in Neanderthals PLoS One 8(12) e82261.

23. Arensburg B*,* et al. (1989) A Middle Paleolithic Human Hyoid Bone. Nature 338: 758-760.

24. Nowak MA, Plotkin JB, Jansen VAA (2000) The evolution of syntactic communication. Nature 404: 495-498.

25. Dediu D, Levinson SC (2013) On the antiquity of language: the reinterpretation of Neandertal linguistic capacities and its consequences. Front Psychol 4:397.

26. Soressi M*,* et al. (2008) Pech-de-l'Azé I (Dordogne, France): nouveau regard sur un gisement moustérien de tradition acheuléenne connu depuis le XIX siècle In: Jaubert J, Bordes J-G, Ortega I. editors. Les sociétés Paléolithiques du grand Sud-Ouest: nouveaux gisements, nouvelles méthodes, nouveaux résultats*.* Société Préhistorique Française, Mémoire XLVII, Paris,, pp 95-132.

27. Soressi M*,* et al. (2013) Neandertals made the first specialized bone tools in Europe. Proc Natl Acad Sci 110:14186-14190

28. McPherron S*,* et al. (2012) New excavations at the Mousterian of Acheulian site of Abri Peyrony (France). Proceedings of the European Society for the Study of Human Evolution 1:102.

29. Clottes J, Menu M, Walter P (1990) La préparation des peintures magdaléniennes des cavernes ariégeoises. Bull Soc Préhist Française 87:170-192.

30. Chalmin E*,* et al. (2006) Minerals discovered in Paleolithic black pigments by transmission electron microscopy and micro-X-ray absorption near-edge structure. Applied Physics A 83:213-218.

31 Salomon, H., 2009. Les matières colorantes au début du Paléolithique supérieur: sources, transformations et fonctions. Université Bordeaux 1, p. 413.

32. Bodu P*,* et al. (2014) An open-air site from the recent Middle Palaeolithic in the Paris Basin (France): Les Bossats at Ormesson (Seine-et-Marne). Quat Int.in press.

33. Zilhão J*,* et al. (2010) Symbolic use of marine shells and mineral pigments by Iberian Neandertals. Proc Natl Acad Sci 107(3):1023-1028.

34. Peresani M, Vanhaeren M, Quaggiotto E, Queffelec A, d’Errico F (2013) An ochred fossil marine shell from the Mousterian of Fumane Cave. PLoS One 8(7) e68572.

35. Roebroeks W*,* et al. (2012) Use of red ochre by early Neandertals. Proc Natl Acad Sci 109:1889-1894.

36. Cârciumaru M, Ion RM, Niţu EC, Ştefănescu R (2012) New evidence of adhesive as hafting material on Middle and Upper Palaeolithic artefacts from Gura Cheii-Râşnov Cave (Romania). J Archaeol Sci 39:1942-1950.

37. Peresani M, Fiore I, Gala M, Romandini M, Tagliacozzo A (2011) Late Neandertals and the intentional removal of feathers as evidenced from bird bone taphonomy at Fumane Cave 44 ky B.P., Italy. Proc Natl Acad Sci 108:3888-3893.

38. Finlayson C*,* et al. (2012) Birds of a feather: Neanderthal exploitation of raptors and corvids. PLoS One 7(9):e45927.

39. Fiore I, Gala M, Tagliacozzo A (2004) Ecology and subsistence strategies in the eastern Italian Alps during the Middle Palaeolithic. Int J Osteoarchaeology 14:273-286.

40. Morin E, Laroulandie V (2012) Presumed Symbolic Use of Diurnal Raptors by Neanderthals. PLoS One 7(3):e32856-.

41. Dibble HL*,* et al. (2009) A preliminary report on Pech de l'Azé IV, layer 8 (Middle Paleolithic, France). PaleoAnthropology 2009:182-219.

42. Kuhn SL*,* et al. (2009) The early Upper Paleolithic occupations at Üçağızli Cave (Hatay, Turkey). J Hum Evol 56: 87-113.

43. Kuhn SL*,* et al. (2004) The Last Glacial Maximum at Meged Rockshelter, Upper Galilee, Israel. Journal of the Israel Prehistoric Society 34:5-47.

44. Roussel M (2011) Normes et variations de la production lithique durant le Châtelperronien : la séquence de la Grande-Roche-de-la-Plématrie à Quinçay (Vienne) [Standards and variations in lithic production during the Châtelperronian: the sequence of the Grande-Roche-de-la-Plématrie at Quinçay (Vienne)] (Université Paris Ouest, Nanterre).

45. Granger JM, Lévêque F (1997) Parure castelperronienne et aurignacienne : étude de trois séries inédites de dents percées et comparaisons. C R Acad Sci Paris IIa 325: 537-543.

46. Zilhão J (2007) The emergence of ornaments and art: an archaeological perspective on the origins of "behavioral modernity". J. Archaeol Res 15:1-54.

47. d'Errico F, Zilhão J, Baffier D, Julien M, Pelegrin J (1998) Neandertal acculturation in Western Europe? A critical review of the evidence and its interpretation. Curr Anthropol 39:S1-S44.

48. Tsanova T (2008) Les débuts du Paléolithique supérieur dans l’Est des Balkans BAR International Series 1752.

49. White R (2002) Observations technologiques sur les objets de parure. In: Schmider B, editor. L’Aurignacien de la grotte du Renne. CNRS, Paris, pp 257-266.

50. Kuhn SL*,* et al. (2009) The early Upper Paleolithic occupations at Ucagizli Cave (Hatay, Turkey). J Hum Evol 56 :87-113.

51. Zilhão J (2011) Aliens from Outer Time? Why the “Human Revolution” Is Wrong, and Where Do We Go from Here? In: Condemi S, Weniger GC editors. Continuity and discontinuity in the peopling of Europ*e.* Springer New York, pp 331-366.

52. Henry-Gambier D, White RW (2006) Modifications artificielles des vestiges humains de l'Aurignacien ancien de la grotte des Hyènes (Brassempouy-Landes). Quelles significations ? In: Cabrera V, Bernaldo de Quiros F editors. El Centenario de la Cueva de El Castillo: El Ocaso de los Neandertales. Ministerio de Educacion y Ciencia, Madrid, pp 71-88.

53. Vanhaeren M (2002) Les fonctions de la parure au Paléolithique supérieur: de l’individu à l’unité culturelle. PhD Thesis, University Bordeaux I, Bordeaux.

54. Mellars P (1998) Neanderthals, Modern Humans and the Archaeological Evidence for Language. In: Jablonski NG, Aiello LC editors. The origin and diversification of language*.* California Academy of Sciences, Vol 24, pp 89-115.

55. Wynn T, Coolidge FL (2008) A Stone-Age Meeting of Minds. Am Sci 96: 44-51.

56. Jacobs Z, Hayes EH, Roberts RG, Galbraith RF, Henshilwood CS (2013) An improved OSL chronology for the Still Bay layers at Blombos Cave, South Africa. J Archaeol Sci 40(1):579-594.

57. Wadley L (2012) Two ‘moments in time’ during Middle Stone Age occupations of Sibudu, South Africa. Southern African Humanities 24:79-97.

58. Soriano S, Villa P, Wadley L (2007) Blade technology and tool forms in the Middle Stone Age of South Africa: the Howiesons Poort and post-Howiesons Poort at Rose Cottage Cave. J Archaeol Sci 34: 681-703.

59. Villa P, Soriano S, Teyssandier N, Wurz S (2010) The Howiesons Poort and MSA III at Klasies River main site, Cave 1A. J Archaeol Sci 37:630-655.

60. Tribolo C*,* et al. (2013) OSL and TL dating of the Middle Stone Age sequence at Diepkloof Rock Shelter (South Africa): a clarification. J Archaeol Sci 40(9):3401-3411.

61. Guérin G, Murray AS, Jain M, Thomsen KJ, Mercier N (2013) How confident are we in the chronology of the transition between Howieson's Poort and Still Bay? J Hum Evol 64: 314-317.

62. Soressi M (2002) Le Moustérien de tradition Acheuléenne du Sud-Ouest de la France. University Bordeaux 1, Bordeaux.

63. Jöris O (2006) Bifacially backed knives (*Keilmesser*) in the Central European Middle Palaeolithic. In: Goren-Inbar N, Sharon G editors. Axe Age: Acheulian Tool-making from Quarry to Discard. Equinox Publishing London, pp 287-310.

64. Guibert P*,* et al. (2008) Une base de données pour la chronologie du Paléolithique moyen dans le Sud-Ouest de la France. In: Jaubert J, Bordes J-G, Ortega I editors. Les sociétés du Paléolithique dans un Grand Sud-ouest de la France*.* Société Préhistorique Française, Mémoire XLVII, Paris, pp 19-40.

65. Jöris O (2004) Zur chronostratigraphischen Stellung der spätmittelpaläolithischen Keilmessergruppen. Bericht der Römisch-Germanischen Kommission 84:49-153.

66. Binford LR (1985) Human ancestors: Changing views of their behavior. J Anthropol Archaeol 4:292-327.

67. Binford LR (1989) Isolating the transition to cultural adaptations: An organizational approach. In: Trinkaus E, editor. The Emergence of Modern Humans: Biocultural Adaptations in the Later Pleistocene. Cambridge University Press, Cambridge, pp 18-41.

68. Stiner MC (1994) Honor Amongst Thieves: A Zooarchaeological Study of Neandertal Ecology. Princeton University Press, Princeton.

69. Marean CW, Assefa Z (1999) Zooarchaeological Evidence for the Faunal Exploitation Behavior of Neanderthals and Early Modern Humans. Evol Anthropol 8: 22-37.

70. Villa P*,* et al. (2005) New data from Ambrona: closing the hunting versus scavenging debate Quat Int 126-128:223-250.

71. Gaudzinski-Windheuser S, Niven L (2009) Hominin Subsistence Patterns During the Middle and Late Paleolithic in Northwestern Europe. In: Hublin J-J, Richards MP editors. The Evolution of Hominin Diets: Integrating Approaches to the Study of Palaeolithic Subsistence. Springer, Leipzig, pp 99-111.

72. Villa P, Lenoir M (2009) Hunting and Hunting Weapons of the Lower and Middle Paleolithic of Europe. In: Hublin J-J, Richards MP editors. The Evolution of Hominin Diets: Integrating Approaches to the Study of Palaeolithic Subsistence. Springer, Leipzig, pp 59-85, pp 59-85.

73. Schmitt D, Churchill SE, Hylander WL (2003) Experimental Evidence Concerning Spear Use in Neandertals and Early Modern Humans. J Archaeol Sci 30:103-114.

74. Marean CW (2005) From the tropics to the colder climates: contrasting faunal exploitation adaptations of modern humans and Neanderthals. In: d'Errico F, Backwell L editors. From Tools to Symbols. From Early Hominids to Modern Humans. Witwatersrand University Press, Johannesburg, pp 333-371.

75. O'Connell JF (2006) How did Modern Humans displace Neanderthals? Insights from hunter-gatherer Ethnography and Archaeology. In: Conard NJ, editor. When Neanderthals and Modern Humans Met. Kerns Verlag, Tübingen, pp 43-64.

76. Berger TD, Trinkaus E (1995) Patterns of Trauma among the Neandertals. J Archaeol Sci 22: 841-852.

77. Pettitt PB (1997) High Resolution Neanderthals? Interpreting Middle Palaeolithic Intrasite Spatial Data. World Archaeology 29(2):208-224.

78. Trinkaus E (2012) Neandertals, early modern humans, and rodeo riders. J Archaeol Sci 39:3691-3693.

79. van Kolfschoten T (2014) The Palaeolithic locality Schöningen (Germany): A review of the mammalian record. Quat Int 326-327: 469-480.

80. Wynn T, Coolidge FL (2011) The implications of the working memory model for the evolution of modern cognition. Int J Evol Biol 2011: Article ID 741357.

81. Voormolen B (2008) Ancient Hunters, Modern Butchers. Schöningen 13II-4, a Kill-Butchery Site Dating from the Northwest European Lower Palaeolithic. Journal of Taphonomy 6:71-247.

82. De Loecker D (2004) Beyond the Site. The Saalian Archaeological Record at Maastricht-Belvédère (The Netherlands). Analecta Praehistorica Leidensia 35/36:1-300.

83. Villa P (2006) Pursuing Neandertals. The Review of Archaeology 27:10-25.

84. Zieba A, Sitlivy V, Sobczyk K, Kolesnik AV (2008) Raw material exploitation and intra-site spatial distribution at two Late Middle and Early Upper Paleolithic sites in the Krakow region: Piekary IIA and Ksiecia Jozefa. Archaeology, Ethnology and Anthropology of Eurasia 33:46-57.

85. Vallverdú J*,* et al. (2005) Short human occupations in the Middle Palaeolithic level I of the Abric Romani rock-shelter (Capellades, Barcelona, Spain). J Hum Evol 48:157-174.

86. Vallverdú J*,* et al. (2012) Combustion structures of archaeological level O and Mousterian activity areas with use of fire at the Abric Romaní rockshelter (NE Iberian peninsula) Quat Int 247:313–324.

87. Henry D (2012) The palimpsest problem, hearth pattern analysis, and Middle Paleolithic site structure. Quat Int 247:246-266.

88. Speth JD, Meignen L, Bar-Yosef O, Goldberg P (2012) Spatial organization of Middle Paleolithic occupation X in Kebara Cave (Israel): Concentrations of animal bones. Quat Int 247:85-102.

89. Alperson-Afil N, Hovers E (2005) Differential use of space in the Neandertal site of Amud Cave, Israel. Eurasian Prehistory 3:3-22.

90. Oron M, Goren-Inbar N (2014) Mousterian intra-site spatial patterning at Quneitra, Golan Heights. Quat Int in press.

91. Solé A, Allué E, Carbonell E (2013) Hearth-related wood remains from Abric Romaní layer M (Capellades, Spain). J Anthropol Res 69: 535-559.

92. Speth JD (2013) Thoughts about hunting: Some things we know and some things we don't know. Quat Int 297:176-185.

93. Hawkes K, O'Connell JF, Rogers L (1997) The behavioral ecology of modern hunter-gatherers, and human evolution. Trends Ecol Evol 12:29-32.

94. Steguweit L (1999) Die Recken von Schöningen - 400,000 Jahre mit dem Speer Mitteilungsblatt der Gesellschaft für Urgeschichte 8:5-14.

95. Villa P, Soriano S (2010) Hunting weapons of Neanderthals and Early Modern Humans in South Africa: similarities and differences. J Anthropol Res 66:5-38.

96. Roach NT, Venkadesan M, Rainbow MJ, Lieberman DE (2013) Elastic energy storage in the shoulder and the evolution of high-speed throwing in Homo. Nature 498: 483-486.

97. Shaw CN, Hofmann CL, Petraglia MD, Stock JT, Gottschall JS (2012) Neandertal humeri may reflect adaptation to scraping tasks, but not spear thrusting. PLoS One 7(7):e40349.

98. Shea JJ (2009) The ecological impact of projectile weaponry in Late Pleistocene human evolution. In: Hublin J-J, Richards MP editors. The Evolution of Hominin Diets: Integrating Approaches to the Study of Palaeolithic Subsistence. Springer, Leipzig, pp 189-199.

99. Brown KS*,* et al. (2012) An early and enduring advanced technology originating 71,000 years ago in South Africa. Nature 491:590-593.

100. Shea JJ (2006) The origins of lithic projectile point technology: evidence from Africa, the Levant, and Europe. J Archaeol Sci 33:823-846.

101. Shea JJ, Sisk ML (2010) Complex projectile technology and Homo sapiens dispersal into western Eurasia. PaleoAnthropology 2010:100-122.

102. Sisk ML, Shea JJ (2011) The African origin of complex projectile technology: an analysis using tip cross-sectional area and perimeter. Int J Evol Biol 2011:968012.

103. Shea JJ (2009) The Impact of Projectile Weaponry on Late Pleistocene Hominin Evolution. In: Hublin J-J, Richards MP editors. The Evolution of Hominin Diets: Integrating Approaches to the Study of Palaeolithic Subsistence. Springer, Leipzig, pp 189-199.

104. Newman K, Moore MW (2013) Ballistically anomalous stone projectile points in Australia. J Archaeol Sci 40(6):2614-2620.

105. Kelly RL (1995) The Foraging Spectrum. Diversity in Hunter-Gatherer Lifeways. Smithsonian Institution Press, Washington, D.C.

106. Völger G (1972) Die Tasmanier. Versuch einer ethnographischen/historischen Rekonstruktion .Fanz Steiner Verlag, Wiesbaden.

107. Cosgrove R, Pike-Tay A, Roebroeks W (2014) Tasmanian archaeology and reflections on modern human behaviour. In: Dennel R, Porr M editors. Southern Asia, Australia and the Search for Human Origins. Cambridge University Press, Cambridge, pp 175-188.

108. Singer R, Wymer J (1982) The Middle Stone Age at Klasies River Mouth in South Africa. University of Chicago Press, Chicago.

109. Wadley L, Mohapi M (2008) A Segment is not a Monolith: evidence from the Howiesons Poort of Sibudu, South Africa. J Archaeol Sci 35:2594-2605.

110. Lombard M (2008) Finding resolution for the Howiesons Poort through the microscope: micro-residue analysis of segments from Sibudu Cave, South Africa. J Archaeol Sci 35:26-41.

111. Villa P*,* et al. (2012) Border Cave and the beginning of the Later Stone Age in South Africa. Proc Natl Acad Sci 109:13208-13213.

112. d'Errico F, et al (2012) Early evidence of San material culture represented by organic artifacts from Border Cave, South Africa. Proc Natl Acad Sci 109: 13214-13219.

113. Klein RG, Steele TE (2013) Archaeological shellfish size and later human evolution in Africa. Proc Natl Acad Sci 110:14186-14190.

114. Robbins LH, Campbell AC, Brook AG, Murphy ML, Hitchkock RK (2012) The antiquity of the bow and arrow in the Kalahari desert: Bone points from White Paintings rock shelter, Botswana. J Afr Archaeol 10:7-20.

115. Lombard M (2011) Quartz-tipped arrows older than 60 ka: further use-trace evidence from Sibudu, KwaZulu-Natal, South Africa. J Archaeol Sci 38:1918-1930.

116. Lombard M, Pargeter J (2008) Hunting with Howiesons Poort segments: pilot experimental study and the functional interpretation of archaeological tools. J Archaeol Sci 35: 2523-2531.

117. McBrearty S, Bishop L, Plummer TRD, Conard N (1998) Tools underfoot: human trampling as an agent of lithic artifact edge modification. Am Antiq 63:108-130.

118. Villa P, Soressi M (2000) Stone tools in carnivore sites: the case of Bois Roche. J Anthropol Res 56:187-215.

119. Yaroshevich A, Kaufman D, Nuzhnyy D, Bar-Yosef O, Weinstein-Evron M (2010) Design and performance of microlith implemented projectiles during the Middle and the Late Epipaleolithic of the Levant: experimental and archaeological evidence. J Archaeol Sci 37(2):368-388.

120. Douka K, Bergman CA, Hedges REM, Wesselingh FP, Higham TFG (2013) Chronology of Ksar Akil (Lebanon) and implications for the colonization of Europe by anatomically modern humans. PLoS One 8 (9) e72931.

121. Zilhão J (2013) Neandertal-Modern Human contact in Western Eurasia: issues of dates, taxonomy and cultural associations. In: Akazawa T, Nishiaki Y, Aoki K editors. Dynamics of learning in Neanderthals and Modern Humans. Volume 1: Cultural perspectives. Springer, Tokyo, pp 21-57.

122. Banks WE, d'Errico F, Zilhão J (2013) Human–climate interaction during the Early Upper Paleolithic: testing the hypothesis of an adaptive shift between the Proto-Aurignacian and the Early Aurignacian. J Hum Evol 64(1):39-55.

123. Marks AE (1983) Prehistory and Paleoenvironment in the Central Negev, Israel. Southern Methodist University, Dallas.

124. Fox JR (2003) The Tor Sadaf lithic assemblages: A technological study of the Early Upper Palaeolithic in the Wadi al-Hasa. In: Goring-Morris AN, Belfer-Cohen A editors. More than meets the eye: Studies on Upper Palaeolithic diversity in the Near East*.* Oxbow Books, Oxford, pp 80-94.

125. Teyssandier N, Bon F, Bordes JG (2010) Within projectile range. Some thoughts on the appearance of the Aurignacian in Europe. J Anthropol Res 66:209-229.

126. Mishra S, Chauhan N, Singhvi AK (2013) Continuity of Microblade Technology in the Indian Subcontinent Since 45 ka: Implications for the Dispersal of Modern Humans. PLoS One 8(7):e69280.

127. Villa P, Delagnes A, Wadley L (2005) A late Middle Stone Age artifact assemblage from Sibudu (KwaZulu-Natal): comparisons with the European Middle Paleolithic. J Archaeol Sci 32:399-422.

128. McBrearty S (2012) Sharpening the mind. Science 491:531-532.

129. Rink WJ*,* et al. (2001) Electron spin resonance (ESR) and thermal ionization mass spectrometric (TIMS) ^230^Th/^234^U dating of teeth in Middle Paleolithic layers at Amud Cave, Israel. Geoarchaeology 16: 701-717.

130. Valladas H*,* et al. (1999) TL Dates for the Neanderthal Site of the Amud Cave, Israel. J Archaeol Sci 26:259-268.

131. Hildebrand WR, King JH (2012) Distinguishing between darts and arrows in the archaeological record: Implications for technological change in the American West. Am Antiq 77: 789-799.

132. Zilhao J (2013) The Upper Palaeolithic of Europe. In: Renfrew C, Bahn P editors. The Cambridge World Prehistory. Cambridge University Press, Cambridge.

133. Richards MP,Trinkaus E (2009) Isotopic evidence for the diets of European Neanderthals and early modern humans. Proc Natl Acad Sci 106:16034-16039.

134. Salazar-García DC*,* et al. (2013) Neanderthal diets in central and southeastern Mediterranean Iberia. Quat Int 318:3-18.

135. Hockett B (2012) The consequences of Middle Paleolithic diets on pregnant Neanderthal women. Quat Int 264:78-82.

136. Speth JD (2012) Middle Palaeolithic subsistence in the Near East: zooarchaeological perspectives – past, present and future. Before Farming 2012/2:1-45.

137. Sanchis Serra A, Fernández Peris J (2008) Procesado y consumo antrópico de conejo en la Cova del Bolomor (Tavernes de la Valldigna, Valencia). El nivel XVIIc (ca 350 ka). Complutum 19:25-46.

138. Blasco R, Fernández Peris J (2012) A uniquely broad spectrum diet during the Middle Pleistocene at Bolomor Cave (Valencia, Spain). Quat Int 252:16-31.

139. Blasco R, Fernández Peris J (2012) Small and large game: Human use of diverse faunal resources at Level IV of Bolomor Cave (Valencia, Spain). C R Palevol 11:265-282.

140. Cochard D, Brugal J-P, Morin E, Meignen L (2012) Evidence of small fast game exploitation in the Middle Paleolithic of Les Canalettes Aveyron, France. Quat Int 264:32-51.

141. Hardy BL*,* et al. (2013) Impossible Neanderthals? Making string, throwing projectiles and catching small game during Marine Isotope Stage 4 (Abri du Maras, France). Quat Sci Rev 82:23-40.

142. Blasco R, Fernández Peris J (2009) Middle Pleistocene bird consumption at Level XI of Bolomor Cave (Valencia Spain). J Archaeol Sci 36:2213-2223.

143. Blasco R*,* et al. (2013) Environmental availability, behavioural diversity and diet: a zooarchaeological approach from the TD10-1 sublevel of Gran Dolina (Sierra de Atapuerca, Burgos, Spain) and Bolomor Cave (Valencia, Spain). Quat Sci Rev 70:124-144.

144. Lev E, Kislev ME, Bar-Yosef O (2005) Mousterian vegetal food in Kebara Cave, Mt. Carmel. J Archaeol Sci 32:475-484.

145. Madella M, Jones MK, Goldberg P, Goren Y, Hovers E (2002) The exploitation of plant resources by Neanderthals in Amud Cave (Israel): The evidence from Phytolith studies. J Archaeol Sci 29: 703-719.

146. Hardy BL (2010) Climatic variability and plant food distribution in Pleistocene Europe: Implications for Neanderthal diet and subsistence. Quat Sci Rev 29:662-679.

147. Hardy K*,* et al. (2012) Neanderthal medics? Evidence for food, cooking, and medicinal plants entrapped in dental calculus. Naturwissenschaften 99:617-626.

148. Ward S, Gale R, Carruthers,W (2012) Late Pleistocene vegetation reconstruction at Gorham’s Cave. In: Barton RNE, Stringer CB, Finlayson JC editors. Neanderthals in context. A report on the 1995-1998 excavations at Gorham’s and Vanguard Caves, Gibraltar. Oxford University School of Archaeology, Oxford, pp 89-101.

149. Henry AG, Brooks AS, Piperno DR (2011) Microfossils in calculus demonstrate consumption of plants and cooked foods in Neanderthal diets (Shanidar III, Iraq; Spy I and II, Belgium). Proc Natl Acad Sci 108:486-491.

150. Cassoli PF, Tagliacozzo A (1997) Avifauna e ittiofauna di Grotta di Castelcivita: considerazioni ecologiche e inquadramento crono-stratigrafico. In: Gambassini P, editor. Il Paleolitico di Castelcivita, culture e ambiente. Electa Napoli, Napoli.

151. Hardy BL, Moncel M-H (2011) Neanderthal Use of Fish, Mammals, Birds, Starchy Plants and Wood 125-250,000 Years Ago. PLoS One 6(8):e23768.

152. Bocherens H, Baryshnikov G, Van Neer W (2014) Were bears or lions involved in salmon accumulation in the Middle Palaeolithic of the Caucasus? An isotopic investigation in Kudaro 3 cave. Quat Int in press.

153. Kvavadze E*,* et al. (2009) 30,000-Year-Old Wild Flax Fibers. Science 325: 1359.

154. Adovasio JM*,* et al. (2001) Perishable industries from Dolní Věstonice I: New insights into the nature and origin of the Gravettian. Archaeology, Ethnology and Anthropology of Eurasia 2(6):48-65.

155. Stiner MC, Munro ND, Surovell TA, Tchernov E, Bar-Yosef O (1999) Paleolithic Population Growth Pulses Evidenced by Small Animal Exploitation. Science 283:190-194.

156. Wadley L (2010) Were snares and traps used in the Middle Stone Age and does it matter? A review and a case study from Sibudu, South Africa. J Hum Evol 58:179-192.

157. McBrearty S, Brooks A (2000) The revolution that wasn't: a new interpretation of the origin of modern human behavior. J Hum Evol 39:453-563.

158. Whallon R (1989) Elements of cultural change in the later Palaeolithic. In: Mellars P, Stringer C editors. The human revolution: Behavioural and biological perspectives on the origin of modern humans. Edinburgh University Press, Edinburgh, Vol 1, pp 433-454.

159. Gamble CS (1999) The Palaeolithic Societies of Europe. Cambridge University Press, Cambridge.

160. Roebroeks W, Kolen J, Rensink E (1988) Planning depth, anticipation and the organization of Middle Palaeolithic technology: the 'archaic natives' meet Eve's descendants. Helinium 28:17-34.

161. Floss H (1994) Rohmaterialversorgung im Paläolithikum des Mittelrheingebietes. Habelt, Bonn.

162. Féblot-Augustins J (1993) Mobility strategies in the late Middle Palaeolithic of central Europe and western Europe: Elements of stability and variability. J Anthropol Archaeol 12:211-265.

163. Féblot-Augustins J (1997) La circulation des matières premières au paléolithique. ERAUL 75, Liège.

164. Féblot-Augustins J (2008) Paleolithic Raw Material Provenance Studies. In: Pearsall DM, editor. Encyclopedia of Archaeology. Academic Press, New York, Vol 2, pp 1187-1198.

165. Féblot-Augustins J (2009) Revisiting European Upper Paleolithic raw material transfers: the demise of the cultural ecological paradigm? In: Adams B, Blades BS editors. Lithic Materials and Paleolithic Societies. Blackwell Publishing, pp 25-46.

166. Adler DS, Barz G, Belfer-Cohen A, Bar-Yosef O (2006) Ahead of the Game: Middle and Upper Palaeolithic Hunting Behaviors in the Southern Caucasus. Curr Anthropol 47:89-118.

167. Deacon HJ (1989) Late Pleistocene palaeoecology and archaeology in the southern Cape, South Africa. In: Mellars PA, Stringer C editors. The human revolution. Edinburgh University Press, Edinburgh, pp 547-564.

168. Wurz S (1999) The Howiesons Poort at Klasies River - an argument for symbolic behaviour. South African Archaeological Bulletin 54:38-50.

169. Ambrose SH (2012) Obsidian dating and source exploitation studies in Africa: Implications for the evolution of human behavior. In: Liritzis I, Stevenson CM Obsidian and ancient manufactured glasses. University of New Mexico Press, Albuquerque, pp 56-74.

170. Slimak L, Giraud Y (2007) Circulations sur plusieurs centaines de kilomètres durant le Paléolithique moyen. Contribution à la connaissance des sociétés néandertaliennes. C R Palevol 6: 359-368.

171. Féblot-Augustins J (1997) Middle and Upper Palaeolithic raw material transfers in western and central Europe: assessing the pace of change. Journal of Middle Atlantic Archaeology 13: 57-90.

172. Féblot-Augustins J (1999) Raw material transport patterns and settlement systems in the European Lower and Middle Palaeolithic: continuity, change and variability. In: Roebroeks W, Gamble CS The Middle Palaeolithic Occupation of Europe. University of Leiden, Leiden, pp 193-214.

173. Valoch K, editor (1988) Die Erforschung der Kulna-Höhle 1961-1976. Anthropos Institut, Brno.

174. Nash DJ*,* et al. (2013) Provenancing of silcrete raw materials indicates long-distance transport to Tsodilo Hills, Botswana, during the Middle Stone Age. J Hum Evol 64:280-288.

175. Wadley L, Kempson H (2011) A review of rock studies for archaeologists and an analysis of dolerite and hornfels from the Sibudu area, KwzZulu-Natal. Southern African Humanities 23:87-107.

176. Minichillo T (2006) Raw material use and behavioral modernity: Howiesons Poort lithic foraging strategies. J Hum Evol 50:359-364.

177. Villa P, Soressi M, Henshilwood CS, Mourre V (2009) The Still Bay points of Blombos Cave (South Africa). J Archaeol Sci 36:441-460.

178. Porraz G*,* et al. (2013) Technological successions in the Middle Stone Age sequence of Diepkloof Rock Shelter, Western Cape, South Africa. J Archaeol Sci 40:3376-3400.

179. Mellars P, French JC (2011) Tenfold Population Increase in Western Europe at the Neandertal-to-Modern Human Transition. Science 333:623-627.

180. Dogandžić T, McPherron SP (2013) Demography and the demise of Neandertals: A comment on ‘Tenfold population increase in Western Europe at the Neandertal-to-modern human transition’. J Hum Evol 64(4):311-313.

181. Mellars P, French JC (2013) Population changes across the Neanderthal-to-modern-human transition in western France: A reply to Dogandžić and McPherron J Hum Evol 65:330-333.

182. Conard NJ, Bolus M, Münzel SC (2012) Middle Paleolithic land use, spatial organization and settlement intensity in the Swabian Jura, southwestern Germany. Quat Int 247:236-245.

183. Turq A, Roebroeks W, Bourguignon L, Faivre J-P (2013) The fragmented character of Middle Palaeolithic stone tool technology. J Hum Evol 65:641-655.

184. Boëda É*,* et al. (2008) Middle Palaeolithic bitumen use at Umm el Tlel around 7000 BP. Antiquity 82:853-861.

185. Holliday TW (1997) Postcranial evidence of cold adaptation in European Neandertals. Am J Phys Anthropol 104:245-258.

186. Smith F (2013) The fate of the Neanderthals. J Anthropol Res 69:167-100.

187. Roebroeks W, Villa P (2011) On the earliest evidence for habitual use of fire in Europe. Proc Natl Acad Sci 108: 5209-5214.

188. Costa A*,* et al. (2012) Quantifying volcanic ash dispersal and impact of the Campanian Ignimbrite super-eruption. Geophysical Research Letters 39 :L10310.

189. Fedele FG, Giaccio B, Hajdas I (2008) Timescales and Cultural Process at 40,000 BP in the Light of the Campanian Ignimbrite Eruption, Western Eurasia. J Hum Evol 55: 834-857.

190. Morley MW, Woodward JC (2011) The Campanian Ignimbrite (Y5) tephra at Crvena Stijena Rockshelter, Montenegro. Quat Res 75: 683-696.

191. Giaccio B*,* et al. (2008) The Campanian Ignimbrite and Codola tephra layers: Two temporal/stratigraphic markers for the Early Upper Palaeolithic in southern Italy and eastern Europe. Journal of Volcanology and Geothermal Research 177: 208-226.

192. Lowe J*,* et al. (2012) Volcanic ash layers illuminate the resilience of Neanderthals and early modern humans to natural hazards. Proc Natl Acad Sci 109:13471-13472.

193. Ambrose SH (1998) Late Pleistocene human population bottlenecks, volcanic winter, and differentiation of modern humans. J Hum Evol 34:623-651.

194. Rampino MR, Ambrose SH (2000) Volcanic winter in the Garden of Eden: The Toba supereruption and the late Pleistocene human population crash. Geological Society of America Special Papers 345:71-82.

195. Ambrose SH (2002) Small things remembered: Origins of early microlithic industries in Sub-Saharan Africa. In: Elston RG, Kuhn S L editors. Thinking small: global perspectives on microlithization*.* Archaeological Papers of the American Anthropological Association*,* Arlington, Virginia, Vol 12, pp. 9-29.

196. Gathorne-Hardy FJ, Harcourt-Smith WEH (2003) The super-eruption of Toba, did it cause a human bottleneck? J Hum Evol 45:227-230.

197. Lane CS, Chorn BT, Johnson TC (2013) Ash from the Toba supereruption in Lake Malawi shows no volcanic winter in East Africa at 75 ka. Proc Natl Acad Sci 110: 8025-8029.

198. Petraglia M et al. (2007) Middle Paleolithic assemblages from the Indian subcontinent before and after the Toba super-eruption. Science 317:114-116.

199. Ambrose SH (2013) Consequences of the Toba super-eruption for human adaptation and evolution. Society for American Archaeology 78th Annual Meeting Abstract 131.
