# Supplementary material for: Neandertal Demise: An Archaeological Analysis of the Modern Human Superiority Complex
Source: PLoS One. 2014 Apr 30;9(4):e96424. doi: 10.1371/journal.pone.0096424 (PMC4005592; doi:10.1371/journal.pone.0096424)
Supplement: Text S2 — A single package of modern behavior? (DOC) [file pone.0096424.s002.doc]

**Neandertal Demise: An Archaeological Analysis of the Modern Human Superiority Complex**

Paola Villa, Wil Roebroeks

**Supporting Information**

**Text S2. A single package of modern behavior?**

The Upper Paleolithic record of Europe has often been treated as a yardstick for modern human behavior, with its characteristics translated into a trait list used to identify the presence of modern human behavioral capacities . In actual fact contemporary modern humans outside of Europe created records which were often very different from the European ones. The limitations of an “Eurocentric (or rather Western European) outlook on cultural evolution” [2] have been stressed many times and for various regions, including the Levant [2], China and North America , and especially by students of the wider Australian record . As detailed by O’Connell and Allen [6, 7], the first occupants of Sahul were modern humans, seafaring populations, who had crossed Wallacea and successfully colonized New Guinea, the Bismarck Archipelago and Australia in probably less than five thousand years. This colonization entailed the production of boats and the development of sophisticated seafaring capacities, generally seen as an unambiguous reflection of “modern” behavior . Nevertheless, over the next twenty thousand years after first landfall in Australia, the descendants of these colonists produced a record with very few indices of ‘modernity’, very different from their contemporaries in western Europe, with for instance almost no evidence of complex lithic and organic technology . The Tasmanian record is particularly relevant in this context [9, 10]. Tasmanian Aboriginal stone technology is similar to the European Middle Paleolithic, but lacks its stone points and Levallois technology, and is mainly composed of multi- and single platform cores, primary flakes, retouched flakes and various sized scrapers . In contrast to the Neandertal Middle Paleolithic record, there is also no direct archeological evidence of hafting or the use of resins or mastics for gluing. The Tasmanian Pleistocene record does not have blades, microliths, hafted bone tools, and carved bone ornaments, no stone lined-hearths and strict spatial organization of activities, and there is little if any technological change occurring for at least 25,000 years. Nonetheless, the first Tasmanians were AMH, as were all occupants of Sahul. What these records clearly show is that the models used to describe human groups as either ‘archaic’ or ‘modern’ are flawed. There is no single package of modern human behavior , with the variability of the archeological record of AMH being larger than usually acknowledged. The factors possibly underlying that variability are debated, but demography is often seen as a contributing one .

**References**

1. McBrearty S, Brooks A (2000) The revolution that wasn't: a new interpretation of the origin of modern human behavior. J Hum Evol 39:453-563.

2. Belfer-Cohen A, Hovers E (2010) Modernity, Enhanced Working Memory, and the Middle to Upper Paleolithic Record in the Levant. Curr Anthropol 51(S1):S167-S175.

3. Bar-Yosef O,Wang Y (2012) Paleolithic Archaeology in China. Annu Rev Anthropol 41: 319-335.

4. Speth JD (2004) News Flash: Negative Evidence Convicts Neanderthals of Gross Mental Incompetence. World Archaeology 36: 519-526.

5. Habgood PJ, Franklin NR (2008) The revolution that didn't arrive: A review of Pleistocene Sahul. J Hum Evol 55:187-222.

6. O'Connell JF, Allen J (2007) Pre-LGM Sahul (Pleistocene Australia-New Guinea) and the archaeology of early modern humans. In: Mellars P, Boyle K, Bar-Yosef O, Stringer CB editors. Rethinking the Human Revolution. McDonald Institute for Archaeological Research, Cambridge) pp 395-410.

7. O'Connell JF, Allen J (2012) The Restaurant at the End of the Universe: Modelling the Colonisation of Sahul Australian Archaeology 74:5-16.

8. Noble W, Davidson I (1996) Human Evolution, Language and Mind.Cambridge University Press, Cambridge.

9. Cosgrove R, Pike-Tay A, Roebroeks W (2014) Tasmanian archaeology and reflections on modern human behaviour. In: Dennel R, Porr M editors. Southern Asia, Australia and the Search for Human Origins. Cambridge University Press, Cambridge, pp 175-188.

10. Holdaway S, Cosgrove R (1997) The archaeological attributes of behaviour: difference or variability? Endeavour 21(2):66-71.

11. Jones R (1977) The Tasmanian paradox. In: Wright RVS Stone tools as cultural markers: change, evolution and complexity. A.I.A.S., Canberra, pp 189-204.
