# Supplementary material for: Neandertal Demise: An Archaeological Analysis of the Modern Human Superiority Complex
Source: PLoS One. 2014 Apr 30;9(4):e96424. doi: 10.1371/journal.pone.0096424 (PMC4005592; doi:10.1371/journal.pone.0096424)
Supplement: Text S3 — Lissoirs. (DOC) [file pone.0096424.s003.doc]

**Neandertal Demise: An Archaeological Analysis of the Modern Human Superiority Complex**

Paola Villa, Wil Roebroeks

**Supporting Information**

**Text S3. Lissoirs**

Archaeologists distinguish bone tools made by percussion flaking, i.e. the same techniques used in knapping stone artifacts, and so-called “formal” bone tools made with techniques specifically conceived for bone and ivory materials, such as splitting and wedging, scraping, grinding and polishing. Bifaces made on elephant bone, such as those found at the Italian Lower Paleolithic sites Castel di Guido and Fontana Ranuccio , are examples of flaked bone tools. Bone and antler projectile points common in Upper Paleolithic sites are examples of formal bone tools.

*Lissoirs* (a French term meaning “smoothers”) are tools made on ungulate ribs, longitudinally split to produce two thin half ribs. These half ribs are then shaped by grinding and scraping, with a rounded end polished by use, showing wear facets and striations. By their similarity to ethnographic bone tools used by the Sami people (Lapps, indigenous people of northern Europe) they are interpreted as tools for smoothing dry hides [2]. They are common in Aurignacian assemblages; for instance, 117 *lissoirs* are reported from three Aurignacian sites in Southwest France (Castanet, Brassempouy and Gatzarria [2]). A few have also been reported in the Protoaurignacian (five in level VII) of Grotte du Renne at Arcy . Twelve “burnishing tools” have been reported in the Châtelperronian level X of Grotte du Renne although by their description only few fall into the category of *lissoirs* . Bone spatulas (with a wider end, common in the Magdalenian) and thicker bone tools made on reindeer antler are often lumped together with *lissoirs* but the bone tools discussed here are all *lissoirs* in a strict sense.

The four *lissoirs* described by Soressi et al. come from two Mousterian of Acheulian Tradition sites, Pech de l’Azė 1 and Abri Peyrony (Southwest France). Three are exactly like Aurignacian *lissoirs*; the fourth (from Abri Peyrony) was on an unsplit rib but its rounded tip was heavily modified and shows spongy bone. All exhibit striations and polish on their rounded end. The Pech de l’Azė 1 tool comes from layer 4, underlying 3 m of undisturbed Middle Paleolithic deposits and is dated by OSL to 51.4 ± 2.0 ka. The three Abri Peyrony tools from two superimposed levels are dated by ^14^C AMS to between 74,710-41,130 cal BP. There are no Upper Paleolithic deposits at both sites. The interpretation of these tools as used for a specific purpose (smoothing of dry hides) is based on their wear and on experimental data. Cultural diffusion from Neandertals to modern humans is a plausible interpretation and supports the idea discussed in the text that some of the innovative technologies of the Protoaurignacian and Aurignacian may have developed out of a Middle Paleolithic base .

**References**

1. Villa P, d'Errico F (2001) Bone and ivory points in the Lower and Middle Paleolithic of Europe. J Hum Evol 41:69-112.

2. Tartar E (2009) De l'os à l'outil. Caractėrisation technique, ėconomique et sociale de l'utilisation de l'os à l'Aurignacien ancien. Universitė Paris 1-Panthėon-Sorbonne, Paris.

3. Julien M, Baffier D, Liolios D (2002) L'outillage en matières dures animales. In: Schmider B, editor*. L'Aurignacien de la Grotte du Renne.* CNRS Editions, Paris, pp 217-249.

4. d'Errico F, Zilhão J, Baffier D, Julien M, Pelegrin J (1998) Neandertal acculturation in Western Europe? A critical review of the evidence and its interpretation. Curr Anthropol 39: S1-S44.

5. Baffier D, Julien M (1990) L'outillage en os des niveaux Châtelperroniens d'Arcy-sur Cure (Yonne). In: Farizy C editor. Palėolithique moyen rėcent et Palėolithique supėrieur ancien en Europe. Musée de Préhistoire d'Ile de France, Nemours, pp 329-334.

6. Soressi M*,* et al. (2013) Neandertals made the first specialized bone tools in Europe. Proc Natl Acad Sci 110:14186-14190.

7. Zilhão J (2007) The emergence of ornaments and art: an archaeological perspective on the origins of "behavioral modernity". J Archaeol Res 15:1-54.
